# Supplementary material for: Turn-on luminescence from molecular rotor realignment in metal-organic framework thin films
Source: Nat Commun. 2026 Mar 14;17:3969. doi: 10.1038/s41467-026-70551-8 (PMC13133237; doi:10.1038/s41467-026-70551-8)
Supplement: Supplementary file 1 — Supplementary Information [file 41467_2026_70551_MOESM1_ESM.pdf]

Supplementary Information

Turn-on Luminescence from Molecular Rotor Realignment in Metal-Organic Framework Thin Films

Jan C. Fischer,[1,7] Tong Zhou,[2,3,7] Philipp Sievers,[4] Nils W. Rosemann,[1,5] Elizabeth Coetsee-Hugo,[6] Dmitry Busko,[1] Yang Li,[1] Honghan Ji,[2,3] Diethelm Johannsmann,[4]* Lingju Guo,[2,3]* Pengfei Duan,[2,3] Bryce S. Richards,[1,5] Ian A. Howard,[1,5,†] and Tonghan Zhao[1]*

[1] Institute of Microstructure Technology, Karlsruhe Institute of Technology, Hermann-von-Helmholtz-Platz 1, 76344 Eggenstein-Leopoldshafen, Germany

[2] National Center for Nanoscience and Technology, No. 11 ZhongGuanCun BeiYiTiao, Beijing 100190, P. R. China

[3] University of Chinese Academy of Sciences, Beijing 100049, P. R. China

[4] Department of Physics, University of the Free State, Bloemfontein 9300, South Africa

[5] Light Technology Institute, Karlsruhe Institute of Technology, Engesserstrasse 13, 76131 Karlsruhe, Germany

[6] Institute of Physical Chemistry, Clausthal University of Technology, Arnold-Sommerfeld-Str. 4, D38678 Clausthal-Zellerfeld, Germany

[7] Jan C. Fischer and Tong Zhou contributed equally to this work.

[†] Present address: Carl Zeiss AG – Innovation Hub Karlsruhe, Hermann-von-Helmholtz-Platz 6, 76344 Eggenstein-Leopoldshafen

Email: johannsmann@pc.tu-clausthal.de; guolj@nanoctr.cn; tonghan.zhao@kit.edu

Content

Supplementary Note 1: Processing method for GIWAXS data and MOF thin film synthesis parameters 4

Supplementary Note 2: *In-situ* QCM and PL measurement 6

Supplementary Note 3: Fluence-dependent Zn-ADC-DABCO PL 7

Supplementary Note 4: Molecular dynamic simulation........ 8

Supplementary Figures........ 9

Supplementary Fig. 1 ATR-FTIR spectra of Zn-ADC-DABCO and ADC. 9

Supplementary Fig. 2 XPS analysis of different elements. 10

Supplementary Fig. 3 Phase structure and orientation confirmed by PXRD. 11

Supplementary Fig. 4 GIWAXS diffractograms of Zn-ADC-DABCO thin films of Si synthesized at different temperatures. 12

Supplementary Fig. 5 GIWAXS diffractograms of Zn-ADC-DABCO thin films on Si made at 40°C from growth solutions with different precursor concentrations as presented in each panel. 13

Supplementary Fig. 6 PXRD and GIWAXS diffactograms of Zn-ADC-DABCO thin films on Si made from different drop-cast growth solution volumes. 14

Supplementary Fig. 7 Estimation of orientation degree. 15

Supplementary Fig. 8 PXRD pattens of Zn-ADC-DABCO film after kept in air and ethanol solution for 5 days. 16

Supplementary Fig. 9 GIWAXS diffractograms of Zn-ADC-DABCO MOF thin film recorded from multiple sample positions. 17

Supplementary Fig. 10 GIWAXS diffractograms of Zn-ADC-DABCO grown on different substrates. 18

Supplementary Fig. 11 GIWAXS diffractograms of Zn-ADC-BPy MOF thin films. 19

Supplementary Fig. 12 FWHM of χ obtained from MTF made by pre-crystallization solution. 20

Supplementary Fig. 13 XPS spectra of MTF-loaded Si and bare Si substrate........ 21

Supplementary Fig. 14 Crystallite thickness over drop-cast growth solution volume........ 22

Supplementary Fig. 15 Possible growth mechanisms leading to preferred MOF thin film orientation during drop-casting on hot plate. 23

Supplementary Fig. 16 GIWAXS diffractograms of thin films made from drop-casting with different linkers. 24

Supplementary Fig. 17 Estimation of activation energy. 25

Supplementary Fig. 18 Zn-ADC-DABCO PL during ethanol evaporation. 26

Supplementary Fig. 19 PL spectrum of ADC in solution. 27

Supplementary Fig. 20 Comparison on PL of oriented MTF and MOF powder deposited on Si substrate. 28

Supplementary Fig. 21 GIWAXS diffractogram of Zn-ADC-DABCO thin film on Si before a) and after b) ethanol dropping experiments. 29

Supplementary Fig. 22 Zn-ADC-BPy PL during methanol evaporation. 30

Supplementary Fig. 23 Absorbance of ADC in MOF thin films. 31

Supplementary Fig. 24 In-situ QCM and PL measurement 32

Supplementary Fig. 25 QCM measurement. 33

Supplementary Fig. 26. QCM and PL measurements with different volumes of liquid ethanol. 34

Supplementary Fig. 27 Time-resolved PL of ADC in solution....................................... 35

Supplementary Fig. 28 Reflectance characteristics of Zn-ADC-DABCO. 36

Supplementary Fig. 29 Reflection of Si substrate at 260 nm during ethanol evaporation. 37

Supplementary Fig. 30 Comparison on absorption of oriented MTF and MOF powder deposited on quartz plate. 38

Supplementary Fig. 31 Simulation of single ethanol molecule passes through MOF. 39

Supplementary Fig. 32 PL evolution of Zn-NDC-DABCO under dropping liquid ethanol. 40

Supplementary Tables 41

Supplementary Table 1 Absolute PLQY of various samples upon excitation of 365 nm. 41

Supplementary Table 2 Fitted lifetimes of ADC PL decay as monomer in solution and in MOF thin film during ethanol evaporation phases. 42

Supplementary Table 3 Tested conditions for preparation of Zn-ADC-DABCO............ 43

Supplementary Table 4 Tested conditions for preparation of Zn-ADC-BPy................... 46

References 47

Supplementary Note 1: Processing method for GIWAXS data and MOF thin film synthesis parameters

Detector images from multiple angles were stitched together based on a projection method, shown previously,[1] resulting in a combined image as if taken by a vertically aligned detector. Further image processing and analysis was performed utilizing the GIXSGUI and GIDVis MATLAB toolboxes,[2, 3] including the representation in reciprocal space coordinates q_z and q_r and the calculation of diffraction patterns based on a bulk crystal structural model from the literature.[4]

In a 2D diffractogram of crystalline thin films, a set of confined intensity maxima indicates a preferred crystallite orientation.[5, 6] The azimuthal width of the diffraction spots, thereby, reflects the angular deviation of individual crystallite orientations from a given texture (resulting in diffraction rings for entirely randomly oriented crystallites).[7] Based on this concept, we assessed the azimuthal full width at half maximum (FWHM) of the {112} diffraction maximum to evaluate the degree of crystallite orientation varying synthesis parameters such as precursor concentration levels, hot plate temperature, and drop volume. As a starting point to evaluate the influence of varying synthesis parameters on the thin film synthesis, we used equimolar ratios for all precursors at a concentration of 0.20 mM and a hot plate temperature of 40°C. We tested different precursor concentrations (keeping the temperature at 40 °C) and hot plate temperatures (keeping all precursors at 0.20 mM). The results of these studies are summarized by the diffractograms in **Supplementary Figs. 4 and 5**.

The hot plate temperature was varied in a range from 30 to 100 °C. The GIWAXS diffractograms of each thereby obtained thin film are shown in **Supplementary Fig. 4**. In order to assess and objectively compare the quality of this preferred orientation across different samples, we quantify the angular misalignment of the DABCO pillar axis inside the MOF with respect to the substrate surface. As a figure of merit, we therefore consider the azimuthal FWHM of the {112} diffraction peak. This is done by Gaussian fitting of the azimuthally integrated intensity after subtracting the background noise taken at a slightly higher radius than that of the {112} peak. The FWHM of this fitted Gaussian serves as an indicator for the orientation quality, whereas we note that this value does not reflect an absolute degree of orientation (which would require reference samples that are fully crystalline and fully amorphous and detailed knowledge about the illuminated thin film volume).[5, 8] The parameter can, however, serve for the comparison of orientational quality across our fabricated sample series. The FWHM values are indicated by insets in the respective diffractograms. According to these numbers, we can conclude that the synthesis yields the highest quality <001>-orientation in a range from 50 to 60 °C. The growth process seems to require a minimum temperature of ~40°C in order to form phase-pure layer pillar MOF structures. The synthesis at 30 °C resulted in a diffraction pattern not even resembling a randomly oriented Zn-ADC-DABCO powder pattern. On the other hand, when the temperature is risen to above 60 °C, the diffraction peaks are smeared out more and more leading to a gradual reduction of the preferred <001>-orientation.

Furthermore, we varied the molar ratio of DABCO with respect to ADC and Zn precursor. We tested a ratio of 1:2 (DABCO 0.10 mM), 1:1 (DABCO 0.20 mM), and 2:1 (DABCO 0.40 mM) and show the hereby obtained GIWAXS diffractograms in **Supplementary Figs 5a to 5c**. From the diffractograms we can conclude that an equimolar ratio of Zn precursor, ADC linker and DABCO pillar is necessary to achieve an oriented thin film, whereas as at DABCO

ratios higher or lower than 1, we observe the formation of an unoriented powder signified by diffraction rings instead of individual points. With this knowledge, the overall concentration level of the precursor solution was investigated next from 0.02 to 0.50 mM, characterized by the resulting diffractograms in **Supplementary Figs. 5d to 5h**, again with the {112} diffraction peak azimuthal FWHM given as a figure of merit for the <001> orientation. We can find a preferential <001>-orientation in all of the samples made from precursor solutions at equimolar ratios. However, apart from the small azimuthal deviation from the <001>-orientation, we observe an additional diffraction peak along the q_z axis at approximately 0.5 $Å^{-1}$ for very low concentrations, (0.02 to 0.1 mM). This peak does not correspond to any of the simulated diffraction peaks and is, therefore, indicating a second, unwanted phase. This reduces the phase-pure synthesis range to precursor concentrations in the growth solution of 0.15 - 0.20 mM, whereby 0.15 mM yields a slightly better-quality preferred orientation.

Taken together, we find that the optimal synthesis parameters for hot drop-casting of Zn-ADC-DABCO are an equimolar precursor concentration of 0.15 mM and a temperature of 50 °C, even though, we note, that within the extend ranges of 0.15 – 0.20 mM and 40 – 60 °C, very similar results can be achieved. In order to get a higher resolution of exactly this parameter range, we performed an additional set of experiments (sweeping the concentration from 0.05 mM to 0.25 mM and the temperature from 45 °C to 60 °C). The combination of these new data points and the ones already acquired leads to the overview in Figure 1e, which clearly reiterates a precursor concentration of 0.15 mM and a temperature of 50 °C yields the highest quality preferential thin film orientation.

We, furthermore, find that this preferred <001>-orientation is present from the first deposition cycle on and independent of the droplet volume. This is evident by the XRD data taken on thin films with different amounts of droplets and different droplet volumes in **Supplementary Fig. 6**. showing the distinct diffraction pattern also in case of only a single applied droplet. In particular, this can be seen by the absence of diffraction peaks other than {002} and its higher order in the case of the PXRD data in **Supplementary Fig. 6a**, and by diffraction spots instead of rings visible in the 2D GIWAXS diffractograms in **Supplementary Figs. 6b and 6c**.

All synthesis parameters for the above-mentioned experiments are summarized in **Supplementary Tables 3** and **4**.

Supplementary Note 2: ***In-situ*** QCM and PL measurement

The Zn-ADC-DABCO MOF thin film was synthesized on the gold-coated quartz crystal (5MHz) resonator by drop-casting method. The evaporation of ethanol was monitored with a QCM instrument with dissipation monitoring. Simultaneously, time-tracking PL spectra were recorded using a spectrometer (AvaSpec-2048L-USB2, Avantes) system with a UV LED (λ = 365 nm) excitation source. The starting time for measurements of QCM and PL were aligned after dropping ethanol. The QCM is an acoustic resonator, which reports the shifts of the resonance frequencies (Δf) and the shifts of the half bandwidth ($\Delta\Gamma$, bandwidth, for short) on a few different overtones. Overtones are labeled by the overtone order n (n = 3, 5, 7, 9, 11 at frequencies of 15, 25, 35, 45, and 55 MHz). $\Delta\Gamma$ is proportional to the shift of the dissipation factor, ΔD. The two are related as $\Delta\Gamma = \Delta D f_{\mathrm{res}}/2$.

When the QCM operates in the gravimetric mode, the overtone-normalized frequency shift, $\Delta f/n$, is negative and is proportional to the mass per unit area, following the Sauerbrey relation.[9] There is no shift in bandwidth. There usually are non-gravimetric contributions to the QCM response, meaning that the shift in bandwidth is nonzero and that the values of $\Delta f/n$ slightly differ between overtones. For planar films, the non-gravimetric response can be turned into a quantitative statement on the film's viscoelasticity.

In order to expose the MTFs to ethanol vapor, the holder was covered with a few sheets of filter paper, which had been soaked in ethanol. The filter paper contained a hole in the center, giving the optics access to the sample. The setup was sealed at the top with a quartz plate. The quartz plate and the filter paper were removed after an exposure time of ~26 minutes (**Supplementary Fig. 24**). The ethanol partial vapor pressure achieved this way was slightly below saturation. The instrument was not temperature-controlled. Had the vapor been truly saturated, droplets would have formed at those places, where the temperature was slightly below the average temperature. Liquid ethanol was not seen.

Supplementary Note 3: Fluence-dependent Zn-ADC-DABCO PL

In the dry state, the linear increase with the fluence means that the number of excitation photons is always directly related to the number of emitted photons from the thin film. In contrast, in the flow state, the sublinear increase in PL intensity with growing exciton density points towards a limiting effect that reduces the number of emitted photons. The fluence-dependent quenching can be explained by exciton-exciton annihilation that becomes more and more significant due to an improved exciton transport among (almost) parallel aligned anthracene units in the flow state.

The nonlinear fit, shown for the flow state PL in Figure 4d, is based on a 1D exciton diffusion model according to Oldenburg et al.[10] for which a fitting parameter $L_{1D}R_0^2$ of ~$1.61*10^{-18}$ cm^3 was found. With an estimated $R_0 = \sqrt[6]{\left(\frac{9*\ln(10)}{6.022*10^{23}*128*\pi^5}\frac{\kappa^2\eta}{n^4}\int I_{PL}(\lambda)*\varepsilon_A(\lambda)*\lambda^4 d\lambda\right)}$ of 3.7 nm based on the extinction coefficient ε_A and emission spectrum I_{PL} of the Zn-ADC-DABCO thin film, an orientational factor κ^2 of 1 for parallel anthracene alignment, a refractive index n of 1.5 and PLQY η of 50 %, this leads to an approximated 1D diffusion length of 120 nm.[11] We conclude a clearly evident change of the excited state dynamics with the rotor alignment inside the MOF thin film.

Supplementary Note 4: Molecular dynamic simulation

All calculations were carried out using the CP2K molecular dynamics package.[12] Structural optimizations were performed using the BFGS algorithm in conjunction with Gaussian-type MOLOPT basis sets (DZVP-MOLOPT-SR-GTH) and GTH-BLYP pseudopotentials.[13] To explore the dynamic pathways of ethanol translocation through the MOF channels, the climbing image nudged elastic band (CI-NEB) method was employed with the extended tight-binding (xTB) force field.[14, 15] To better describe weak intermolecular interactions, DFT-D3(BJ) dispersion corrections were applied.[16, 17] Two simulation models, expanded to $2\times1\times2$ and $2\times2\times2$ supercells based on the unit cell of the Zn-ADC-DABCO layer-pillar MOF, were constructed to investigate the molecular dynamics of ethanol molecules passing through the pores—either individually or as continuous chains from both sides. The two models comprise 972 and 1980 atoms, respectively. To accurately capture the interactions between ethanol molecules and the ADC framework, all atoms were treated as fully flexible during the simulations.

Supplementary Figures

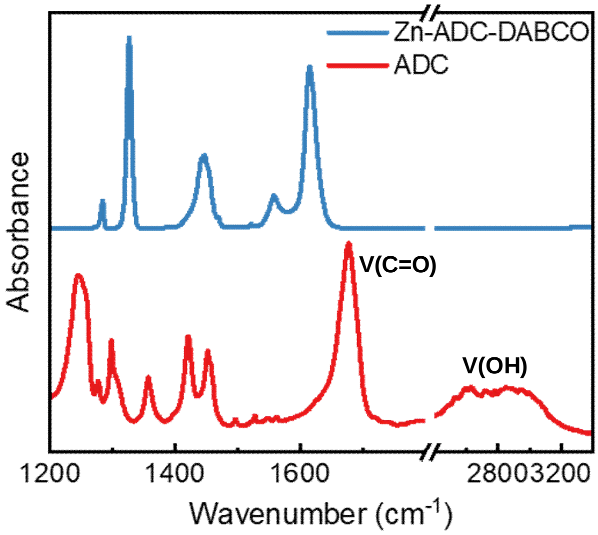

Supplementary Fig. 1 ATR-FTIR spectra of Zn-ADC-DABCO and ADC. The characteristic absorption bands at 1677 cm^{-1} is the stretching vibration of C=O bonds, and the absorption bands at 2500–3100 cm^{-1} are assigned to the carboxylic O–H stretching vibrations in curve of ADC. 1676 cm^{-1} of the stretching vibration of C=O bonds and 2500–3100 cm^{-1} of the carboxylic O–H stretching vibrations are disappeared in curves of Zn-ADC-DABCO. Source data are provided as a Source Data file.

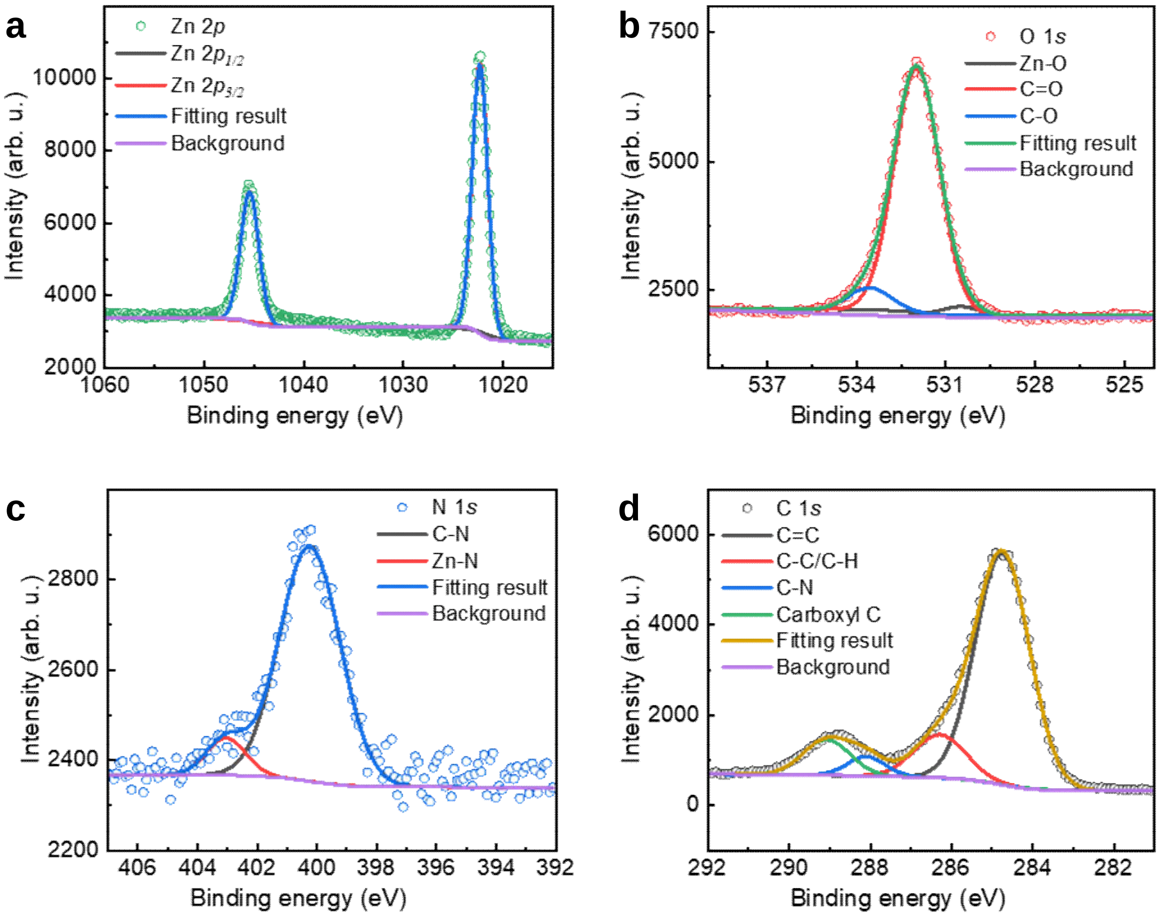

Supplementary Fig. 2 XPS analysis of different elements. (a) Zn 2*p*, (b) O 1*s*, (c) N 1*s*, (d) C 1*s*. Source data are provided as a Source Data file.

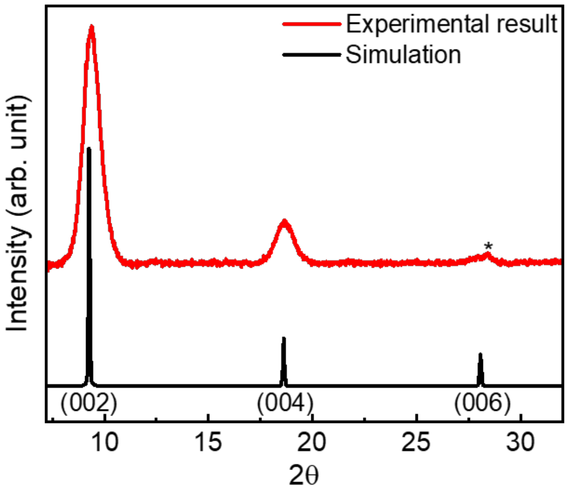

Supplementary Fig. 3 Phase structure and orientation confirmed by PXRD. PXRD pattern of Zn-ADC-DABCO (red line) and simulated diffractogram with preferred orientation along the [001] direction (black line) as reference. The small diffraction peak at ~28.5° (labelled by *) stems from the underlying Si substrate. Source data are provided as a Source Data file.

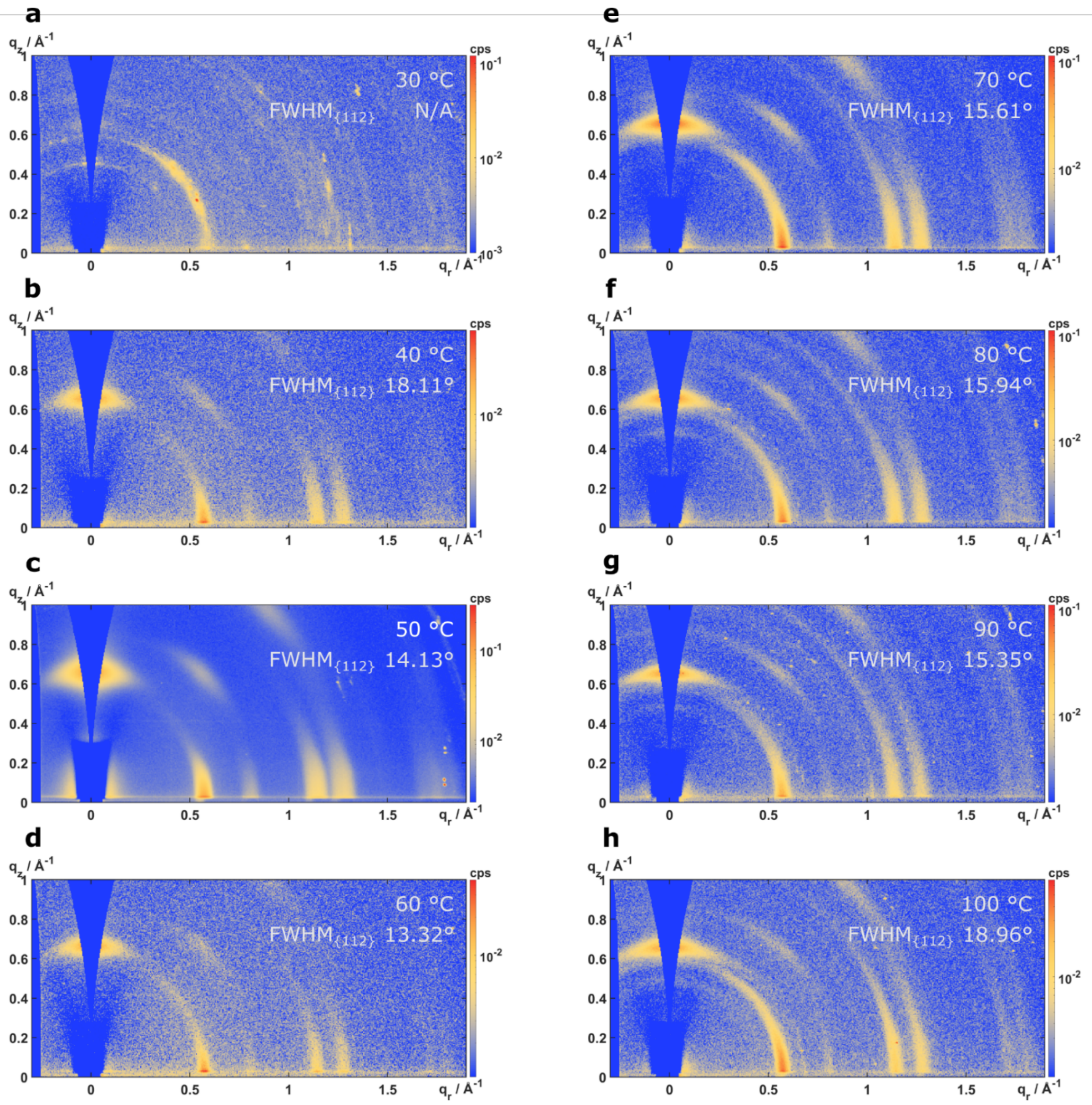

Supplementary Fig. 4 GIWAXS diffractograms of Zn-ADC-DABCO thin films of Si synthesized at different temperatures. a) to h) show the results of temperatures ranging from 30 to 100 °C. The temperature range, in which phase-pure Zn-ADC-DABCO was achieved (without additional peaks along the q_z axis except the <001> peak at ~0.67 Å^{-1}) is 40 to 60 °C. Concluding, additionally, from the quality of the <001>-orientation assessed by the {112} FWHM indicated in each panel, the optimal drop-casting temperature is 50 °C.

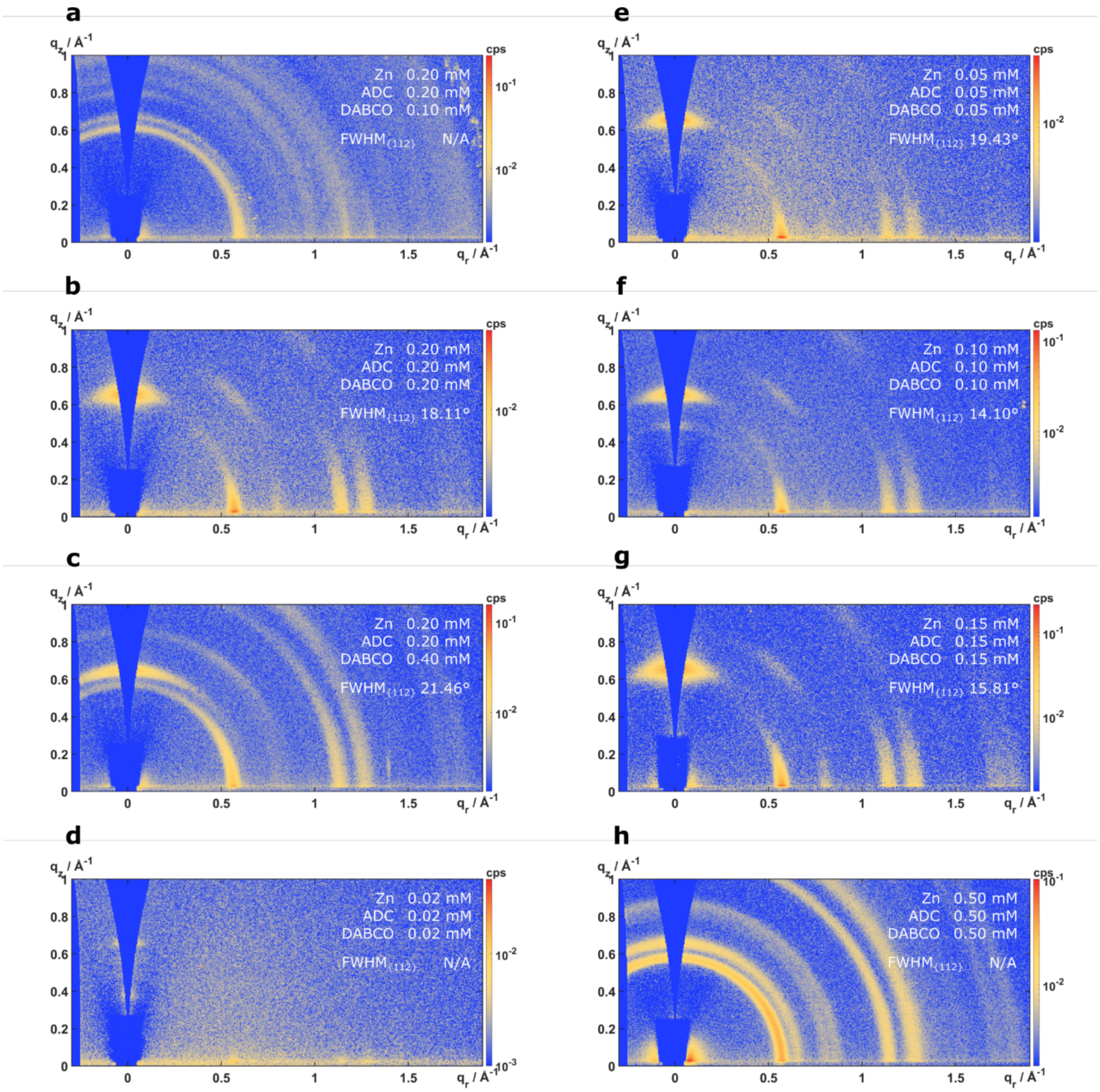

Supplementary Fig. 5 GIWAXS diffractograms of Zn-ADC-DABCO thin films on Si made at 40°C from growth solutions with different precursor concentrations as presented in each panel. For a) to c) high precursor concentrations, diffraction rings are visible indicating a random crystallite orientation on the substrate. For d) to h) lower concentrations, much more confined diffraction maxima hint towards oriented growth on the substrate. However, only the samples made from concentrations above 0.10 mM are observed to be phase-pure Zn-ADC-DABCO. The diffractograms of samples made from 0.10 mM or lower concentrations exhibit another diffraction maximum in the q_z direction at ~0.5 Å^{-1} which is not expected from the Zn-ADC-DABCO lattice. In combination with considering the {112} FWHM to evaluate the quality of the <001>-orientation, this leads to the optimal precursor concentration of 0.15 mM for each component inside the growth solution.

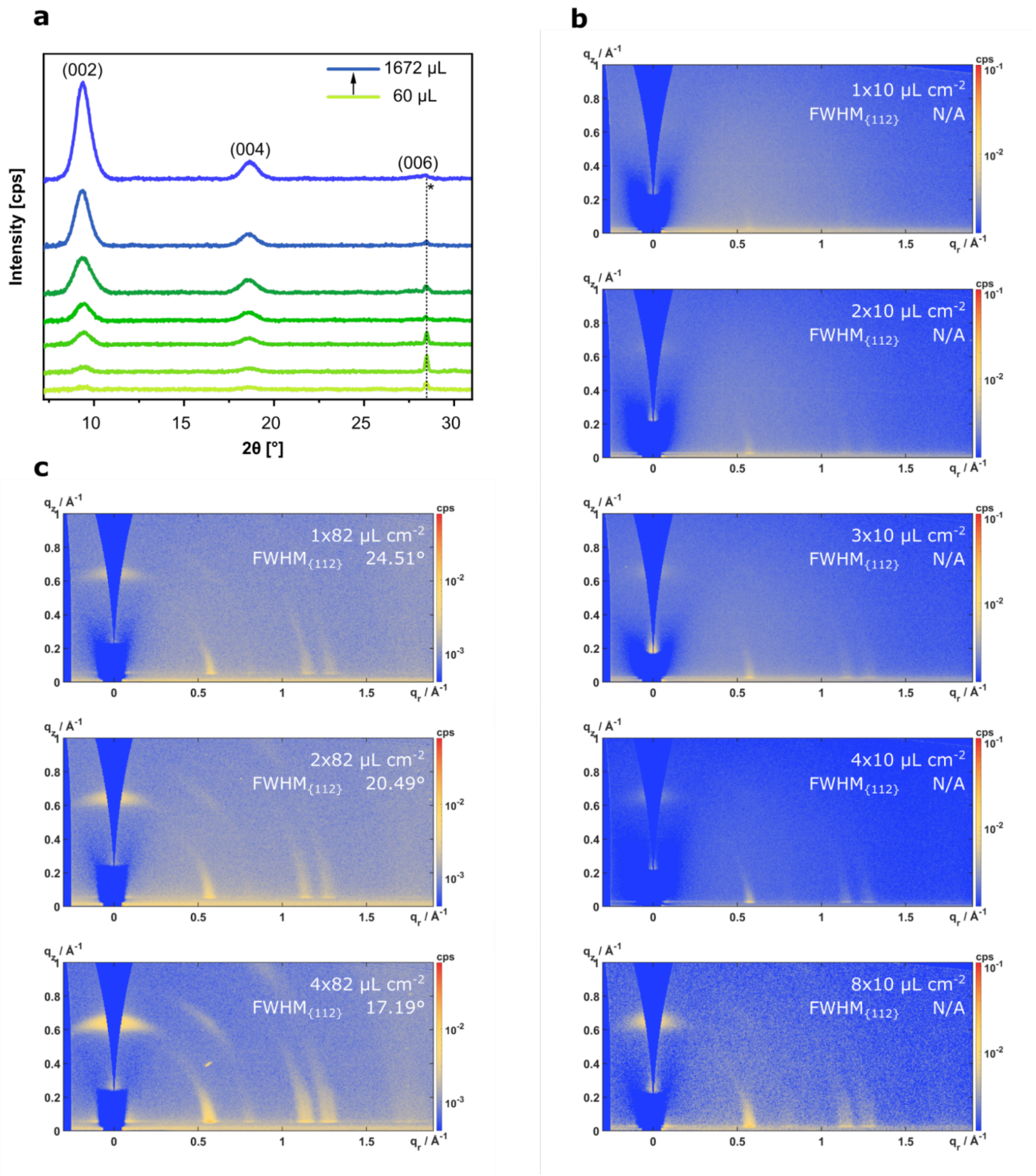

Supplementary Fig. 6 PXRD and GIWAXS diffactograms of Zn-ADC-DABCO thin films on Si made from different drop-cast growth solution volumes. a) PXRD of samples made from 30 uL/cm^2 droplets up to a total volume of 1672 µL. The diffraction patterns show a clear increase of peak intensity with increasing total volume, signifying the growing amount of MOF material on the substrate. Since a PXRD pattern is measured in a Bragg-Brentano geometry, the obtained patterns purely reflect on the horizontal diffraction planes that are related to the DABCO pillar spacing (hence the (002), (004), (006) labeling). The small diffraction peak at ~28.5° (labelled by *) stems from the underlying Si substrate. **b)** and **c)** show GIWAXS diffractograms of samples made from 10 uL/cm^2 and 82 uL/cm^2. The latter droplet volume is the maximum amount that could still be supported by the substrate by surface tension without leaking. Matching the observations from the PXRD data, also the GIWAXS data shows a gradual increase of peak intensities with increasing total drop-cast volume, independent of the volume per droplet. In case of the 82 uL/cm^2 droplet batch, allowing for an assessment of the {112} FWHM, we can observe a stepwise decrease of the fitted Gaussian width which could imply a templating effect of already deposited MOF layers.

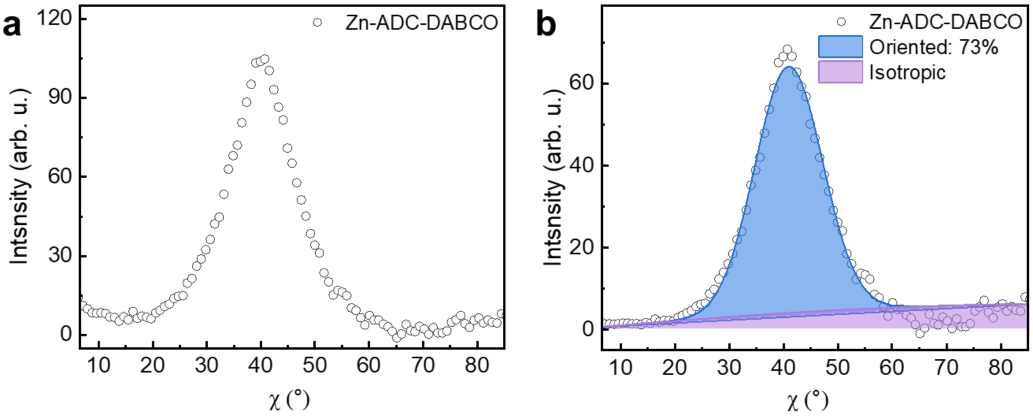

.

Supplementary Fig. 7 Estimation of orientation degree. a) Radial intensity distribution of (112) plane as a function of azimuthal angle χ. b) Orientation degree is estimated by Gaussian peak fitting and integration of peak area. Source data are provided as a Source Data file.

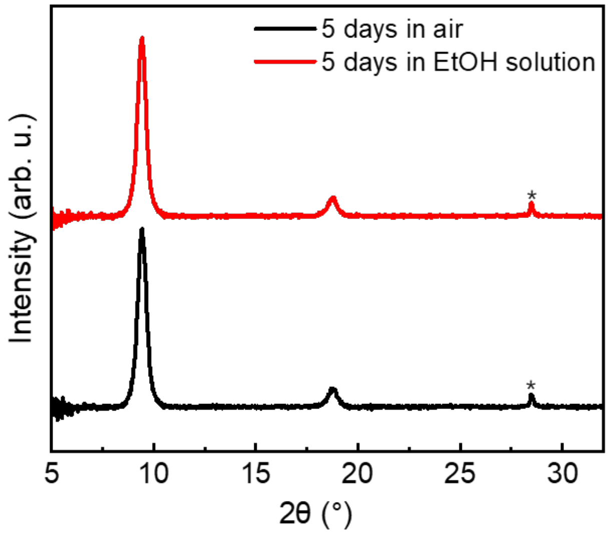

Supplementary Fig. 8 PXRD pattens of Zn-ADC-DABCO film after kept in air and ethanol solution for 5 days. The crystal structure and orientation remain. The small diffraction peak at ~28.5° (labelled by *) stems from the mixing signals of Zn-ADC-DABCO and underlying Si substrate. Source data are provided as a Source Data file.

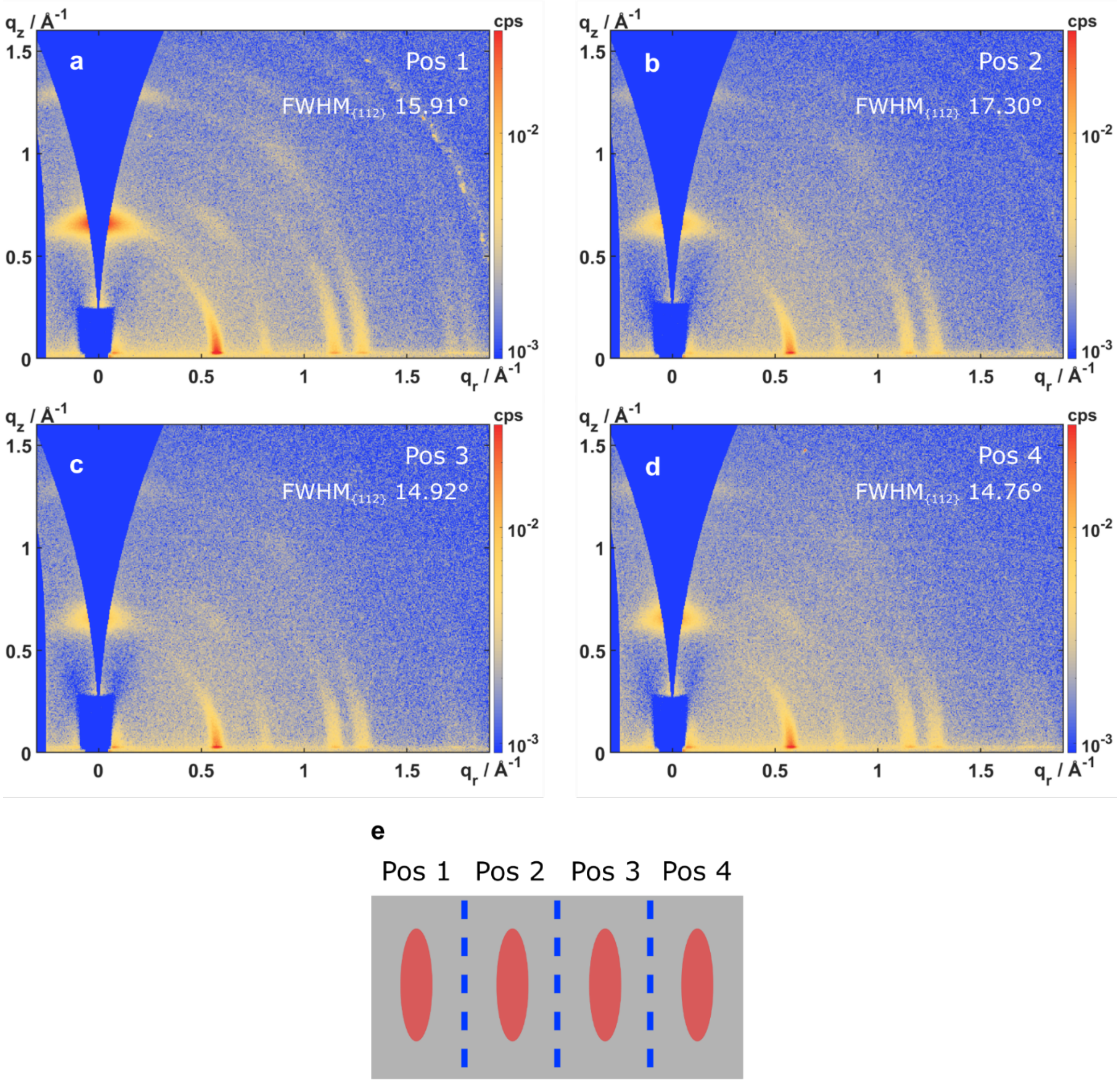

Supplementary Fig. 9 GIWAXS diffractograms of Zn-ADC-DABCO MOF thin film recorded from multiple sample positions. a) to **d)** show the diffractograms taken at the positions 1 to 4 illustrated in **e)**. We can observe good homogeneity across the sample, quantitatively assessed by the similar {112} FWHM values at all measured positions.

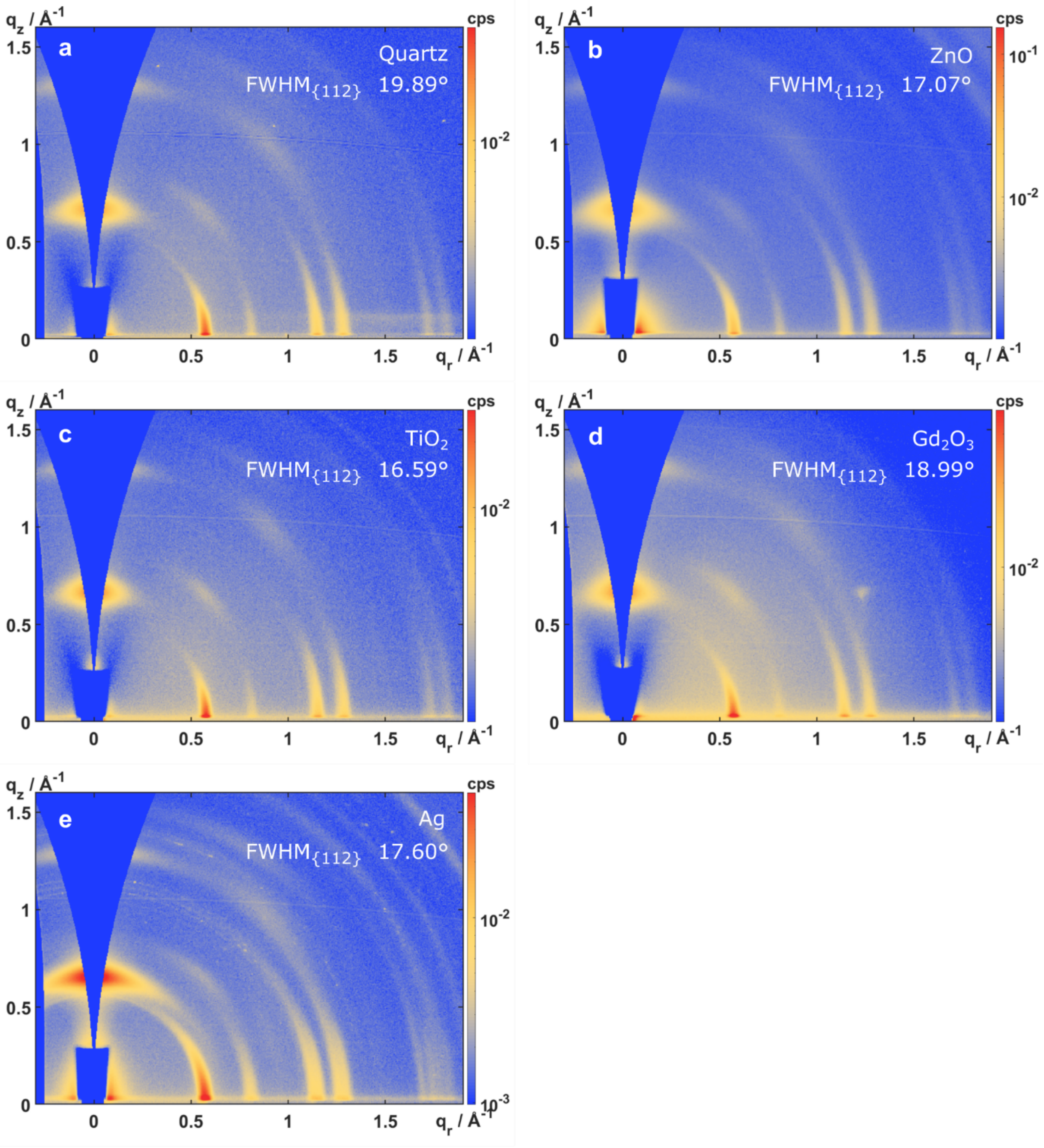

Supplementary Fig. 10 GIWAXS diffractograms of Zn-ADC-DABCO grown on different substrates. These are (a) quartz, (b) ZnO, (c) TiO_2, (d) Gd_2O_3, and e) silver. All diffraction patterns exhibit diffraction maxima comparable to the pattern examined in detail in Fig. 1d, pointing towards a <001>-orientation. Based on the {112} FWHM values as indicated in each panel, the ZnO substrate yielded the best quality of preferred orientation. Although showing a comparable angular deviation from the <001>-orientation as the samples on the other substrates, the film on silver simultaneously shows underlying diffraction rings. These indicate that a significant portion of the thin film in fact was grown randomly oriented. This is evidence suggesting a certain degree of DABCO pillar metal oxide coordination taking place, as all tested oxide substrates exhibit much better confined diffraction peaks.

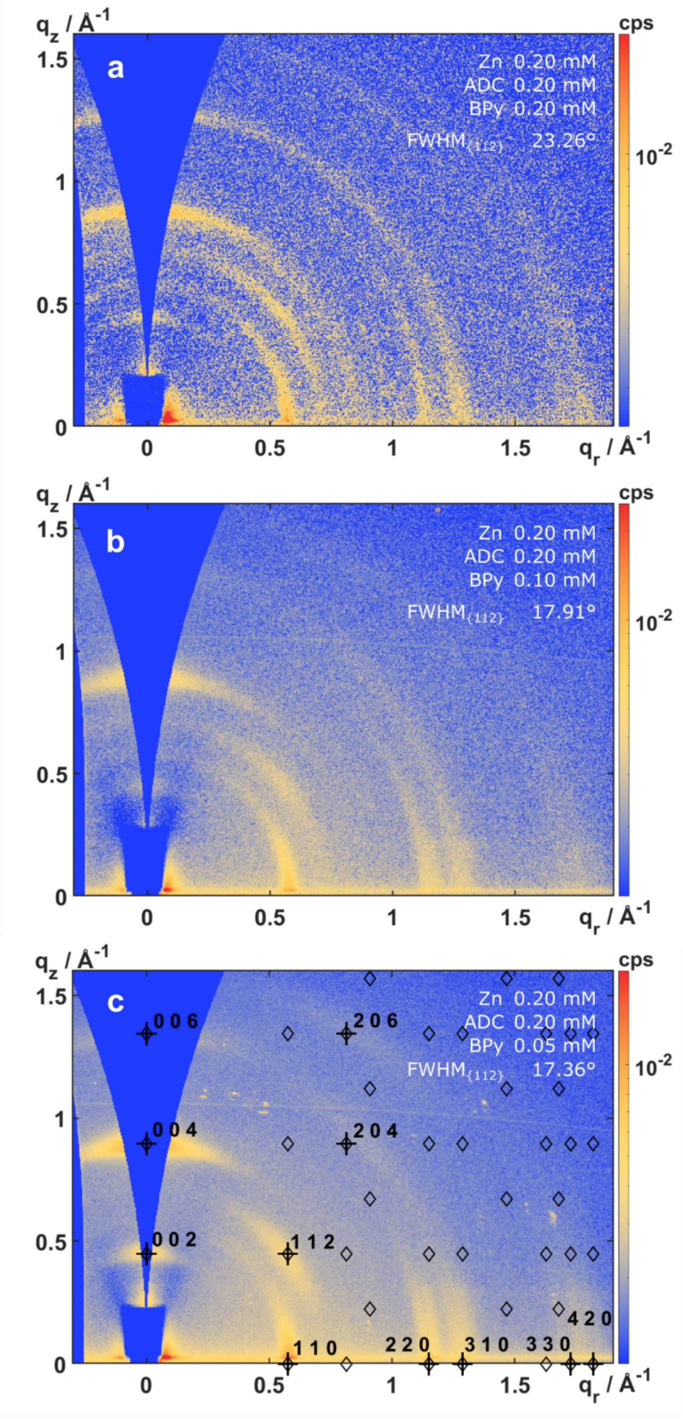

Supplementary Fig. 11 GIWAXS diffractograms of Zn-ADC-BPy MOF thin films. a-c show GIWAXS diffractograms of thin films made by the different concentrations of precursors in the drop-cast growth solutions as indicated in each panel. The combination of Zn and ADC precursor at 0.20 mM and BPy at 0.05 mM resulted in the highest quality preferred orientation judged by the azimuthal FWHM of the {112} diffraction peak, indicated in c. Here, the experimental GIWAXS diffractogram of Zn-ADC-BPy on Si is shown with an overlay of the calculated diffraction peak positions (black diamond shapes) expected from the bulk crystal model.[18] The simulated peak positions at which a matching experimental diffraction maximum is observed, are labeled with their respective Laue indices.

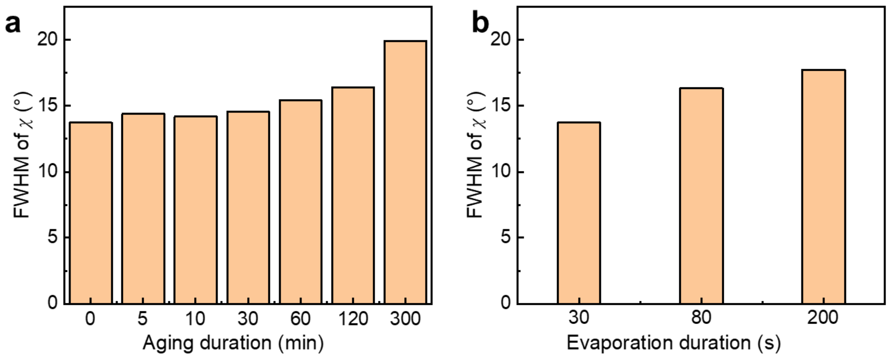

Supplementary Fig. 12 FWHM of χ obtained from MTF made by pre-crystallization solution. a) Aging duration of the precursor solution prior to drop-casting. b) Ethanol evaporation duration during the MTF formation process. Source data are provided as a Source Data file.

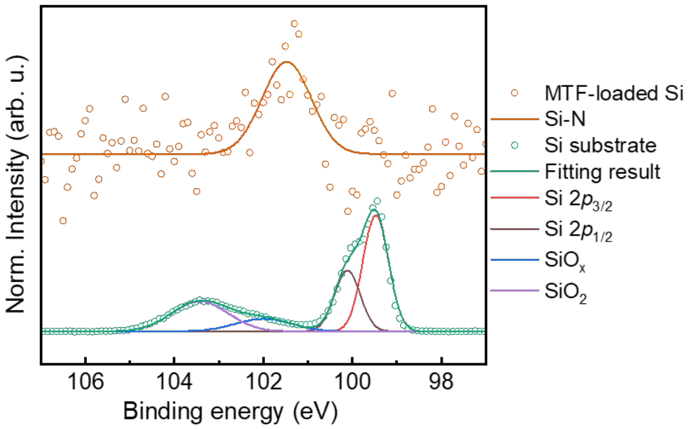

Supplementary Fig. 13 XPS spectra of MTF-loaded Si and bare Si substrate. XPS analysis of the substrate interface reveals a weak peak corresponding to Si-N bonds, suggesting a chemical interaction between the Si surface and the DABCO linker. Source data are provided as a Source Data file.

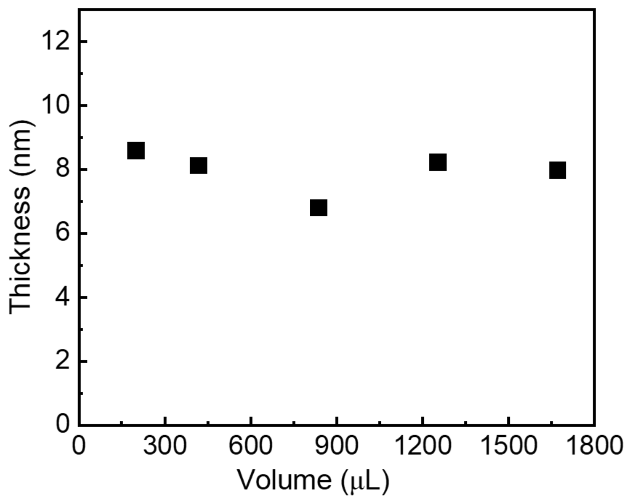

Supplementary Fig. 14 Crystallite thickness over drop-cast growth solution volume. The estimated crystallite size (thickness) is based on the Scherrer equation ($d = K\lambda/(\beta\cos\theta)$, with the Scherrer constant $K \approx 0.9$, the wavelength of X-ray photons $\lambda \approx 0.154$ nm, and the diffraction angle θ) and the FWHM (β, in radians) of the first diffraction peak progression of the PXRD patterns in Supplementary Fig. 6. Source data are provided as a Source Data file.

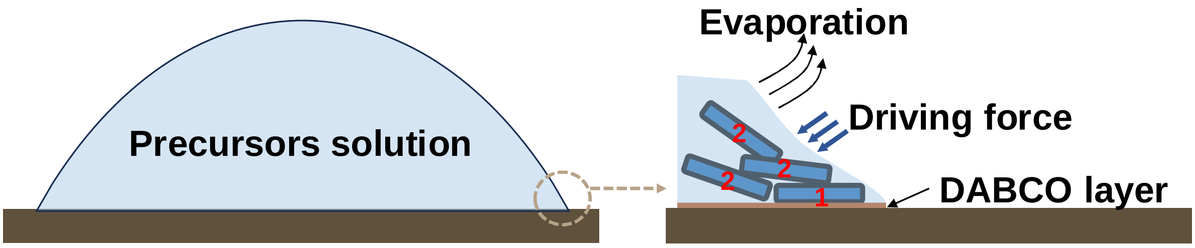

Supplementary Fig. 15 Possible growth mechanisms leading to preferred MOF thin film orientation during drop-casting on hot plate. 1) MOF thin film growth by crystallization from the surface, via the formation of a pillar molecule functional layer on a metal-oxide substrate. 2) MOF thin film growth by formation of nanoplatelets in solution and adopts lying-flat morphology driving by lowest-surface-energy facet and horizontal capillary force. A plate-like crystallize shape, thereby, favors ordered stacking on the substrate surface. As shown by Hupp and co-workers,[19] DABCO molecules can directly interact with a metal-oxide surface enabling surface-initiated MOF film growth with the pillar molecules vertically oriented. Secondly, literature suggests that the bond between Zn paddle wheels and pillar molecules can be attacked by water.[20, 21] However, steric protection by bulky groups of surrounding linkers, such as ADC, can prevent a pillar exchange for water.[21, 22] Based on this mechanism, the formation of the Zn-ADC paddle wheels is a necessary prerequisite for the stable installation of DABCO pillars. Consequently, the MOF exhibits a faster growth rate along the layer linker plane, resulting in MOF platelets which are most likely to lie flat on the substrate. This is also an explanation for the indication of preferred orientation on the silver surface (Supplementary Fig. 10e), which does not offer the possibility of DABCO-anchoring. We suggest that the relative stability of the Zn-ADC paddle wheel is key to allowing this facile fabrication process. Testing the synthesis protocol using successively less bulky linkers 1,4-naphthalene-dicarboxylic acid (NDC) and 1,4-benzene-dicarboxylic acid (BDC) still seems to produce crystalline structures. However, we observe a significant loss of preferred orientation and long-range atomic order the smaller the aromatic central group **(**Supplementary Fig. 16). Further searching and targeted synthesis design could extend the number of linkers that function with this highly attractive and easily scalable fabrication process.

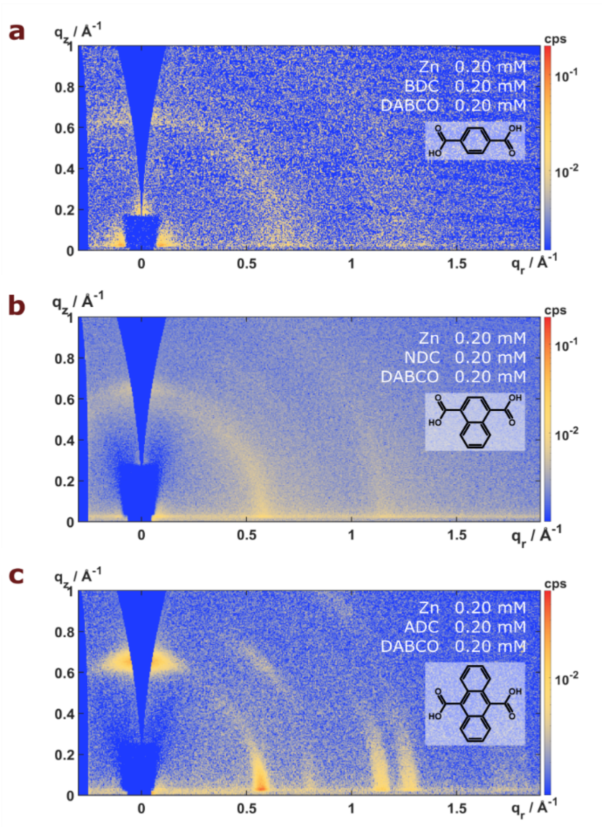

Supplementary Fig. 16 GIWAXS diffractograms of thin films made from drop-casting with different linkers. a Zn-BDC-DABCO. **b** Zn-NDC-DABCO. **c** Zn-ADC-DABCO. All thin films were made with an equimolar ratio of precursors of 0.20 mM and 50 °C hot plate temperature. In contrast to the bulky ADC linker creating a highly oriented thin film, the poorly defined, ring-shaped diffraction patterns of the NDC- and BDC-based samples signal worse crystallinity and no preferred orientation.

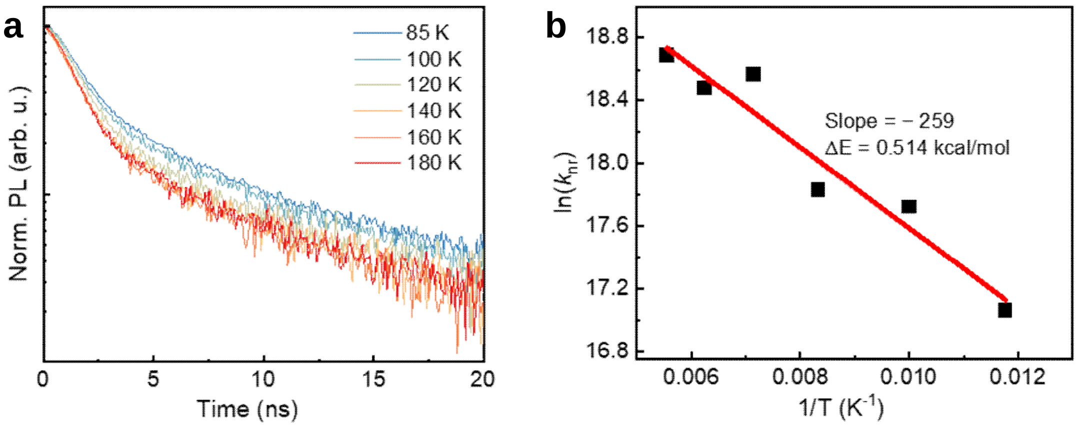

Supplementary Fig. 17 Estimation of activation energy. (a) Temperature-dependent PL lifetime spectra. (b) Activation energy estimation by Arrhenius equation $k_{nr}(T) = A \cdot \exp(-E_a/(k_B T))$, where k_{nr} is non-radiative transition rate, A is pre-exponential factor, E_a is activation energy, k_B is Boltzmann constant. Source data are provided as a Source Data file.

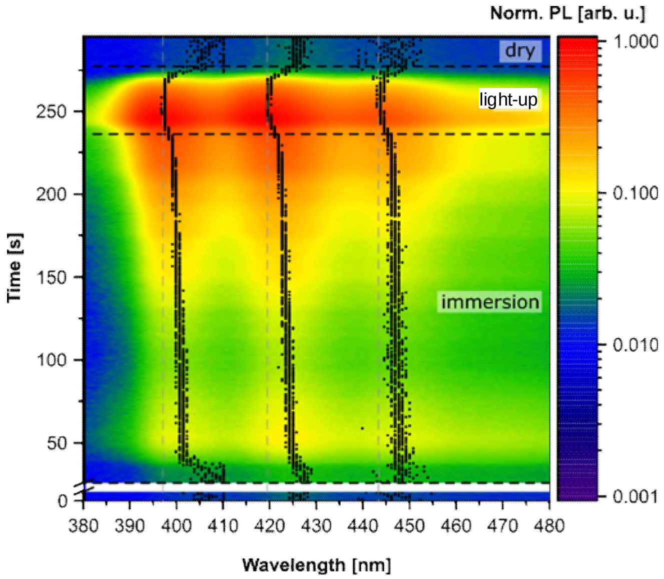

Supplementary Fig. 18 Zn-ADC-DABCO PL during ethanol evaporation. Zoomed-in version of the plot in Fig. 2b, focussing on the region of the three dominant ADC emission peaks. The black dots indicate the peak positions in each individual spectrum, showing significant shifts occurring after the solvent droplet application (clipped region), and in the final evaporation phase, when the last ethanol molecules are leaving the MOF pores shortly before the film turns dry again.

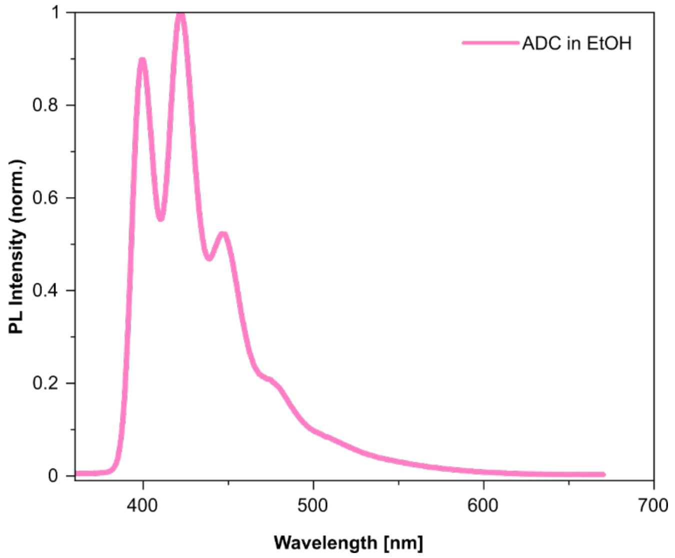

Supplementary Fig. 19 PL spectrum of ADC in solution. The emission of ADC monomer dissolved in ethanol at a concentration of 5×10^{-8} mol L^{-1} shows clear vibronic structure with strong peaks at 399 , 422 , and 447 nm.

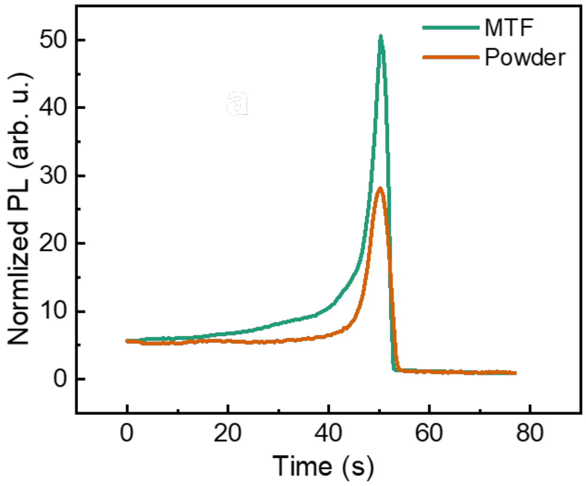

Supplementary Fig. 20 Comparison on PL of oriented MTF and MOF powder deposited on Si substrate. PL intensity (averaged from 410 to 430 nm) over time. Source data are provided as a Source Data file.

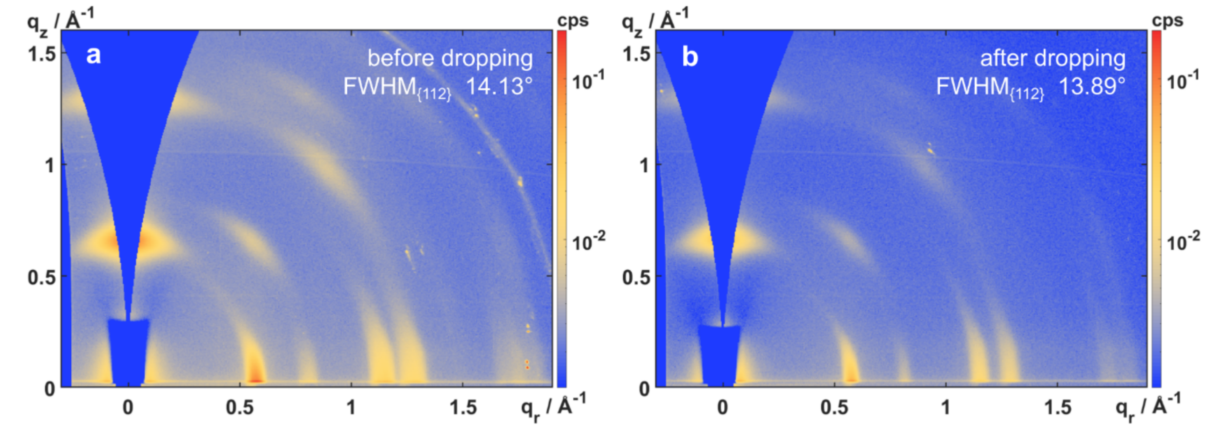

Supplementary Fig. 21 GIWAXS diffractogram of Zn-ADC-DABCO thin film on Si before a) and after b) ethanol dropping experiments. The peak positions remaining the same even after extensive ethanol dropping indicates that the MOF morphology and orientation are not affected by the repeated application and evaporation of the solvent. The slight decrease in signal intensity can be due to slightly different measurement positions of the thin film, which cannot be reproduced exactly between different measurements.

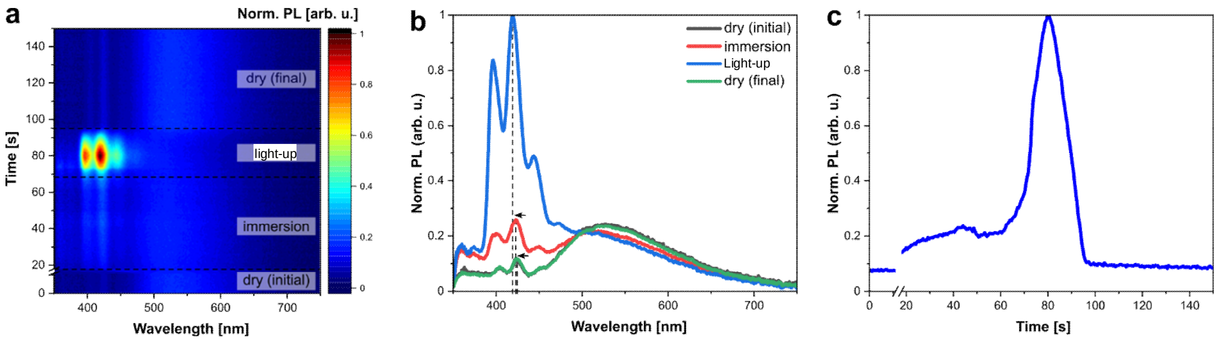

Supplementary Fig. 22 Zn-ADC-BPy PL during methanol evaporation. a) Spectral evolution over time. **b)** Averaged PL intensity according to evaporation phases. **c)** Average PL intensity (410 – 430 nm) over time. The sample was continuously illuminated by a 300 nm LED. Zn-ADC-BPy shows the same characteristic PL turn-on effect as the DABCO-based system with different emission states accompanying the solvent evaporation phases.

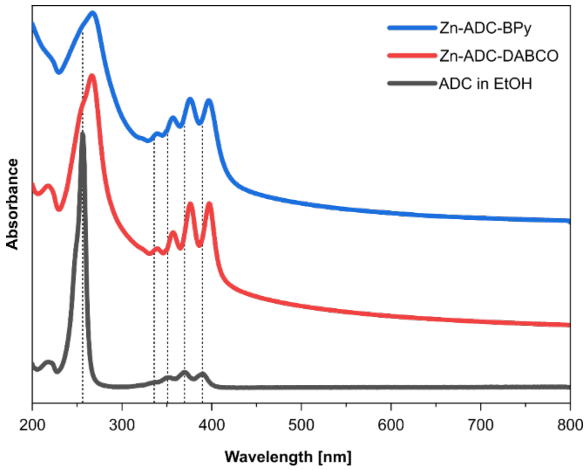

Supplementary Fig. 23 Absorbance of ADC in MOF thin films. Absorbance of ADC in ethanol at a concentration of 5 $\times 10^{-6}$ M is shown comparison to Zn-ADC-DABCO and Zn-ADC-BPy MOF thin films on quartz substrates. The similarity of ADC and MOF thin film spectra indicates the linker as central component governing absorption. The red-shifted appearance of both MOF thin film absorbance peaks with respect to the solvated linker is sign of linker coordination with metal clusters in the MOFs.

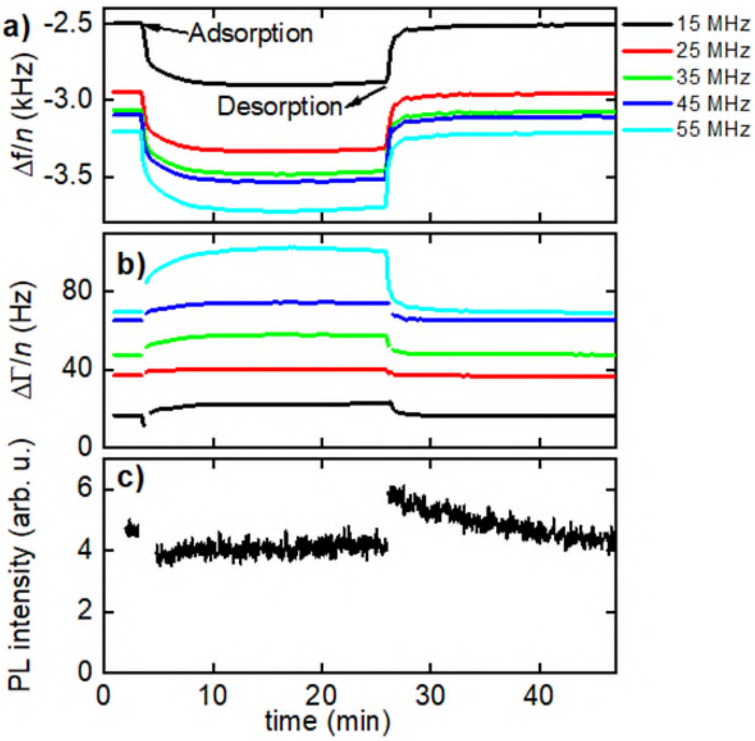

Supplementary Fig. 24 In-situ QCM and PL measurement acquired while sample is exposed to ethanol vapor. (a) Overtone-normalized frequency shifts (b) Overtone-normalized bandwidth shifts, and (c) Evolution of PL versus time. The artifacts from the vapor adding and removing are omitted in the PL signal. Source data are provided as a Source Data file.

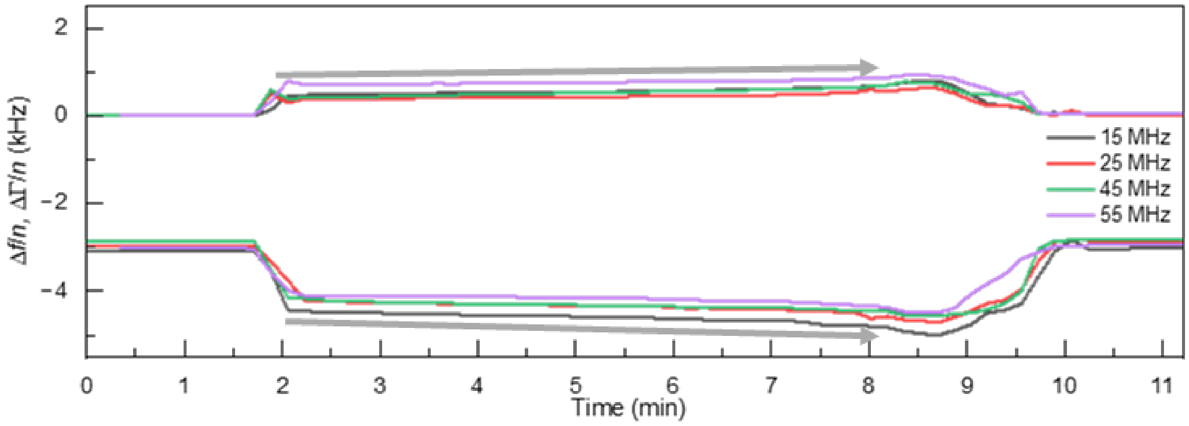

Supplementary Fig. 25 QCM measurement. Shifts in overtone-normalized resonance frequency, Δf/n, and half-bandwidth, ΔΓ/n, upon dropping 20 μL ethanol on the MOF thin film-coated resonator. Source data are provided as a Source Data file.

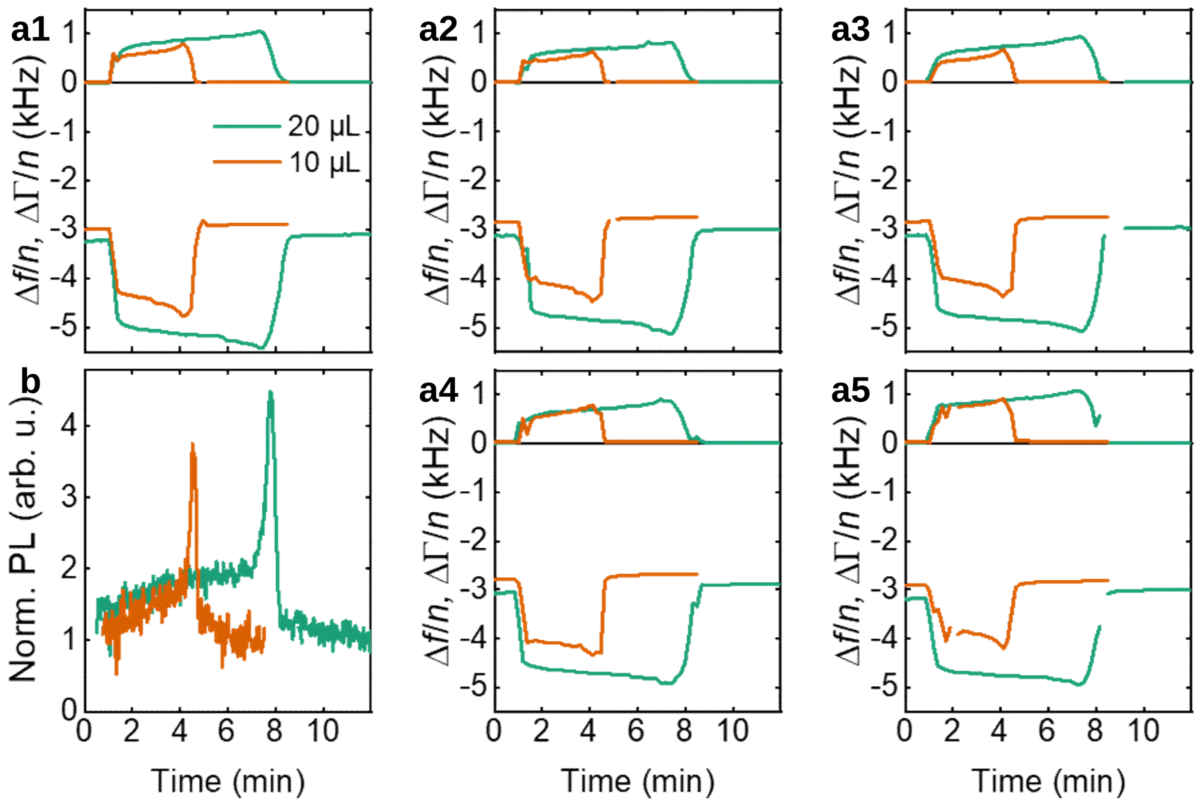

Supplementary Fig. 26. QCM and PL measurements with different volumes of liquid ethanol. (**a1–a5**) Shifts in overtone-normalized resonance frequency, Δf/n (lower), and half-bandwidth, ΔΓ/n (upper), upon dropping 20 μL and 10 μL ethanol on the MOF thin film-coated resonator. For the overtone recorded, **a1**) 15 MHz, **a2**) 25 MHz, **a3**) 35 MHz, **a4**) 45 MHz, **a5**) 55 MHz. (**b**)PL measurement is performed in parallel. Source data are provided as a Source Data file.

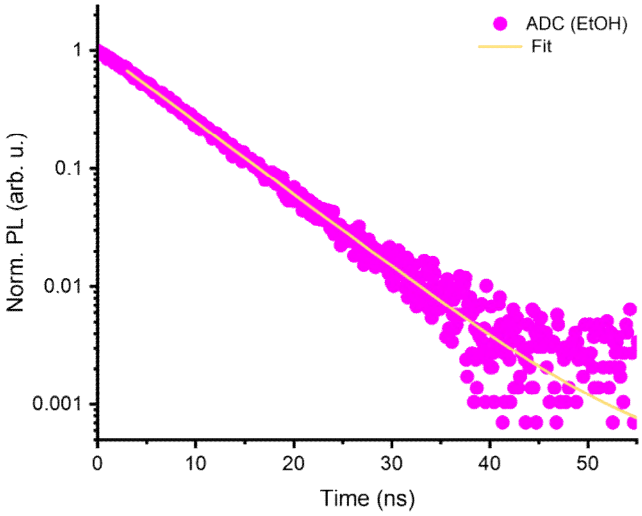

Supplementary Fig. 27 Time-resolved PL of ADC in solution. λ_{ex} = 365 nm.

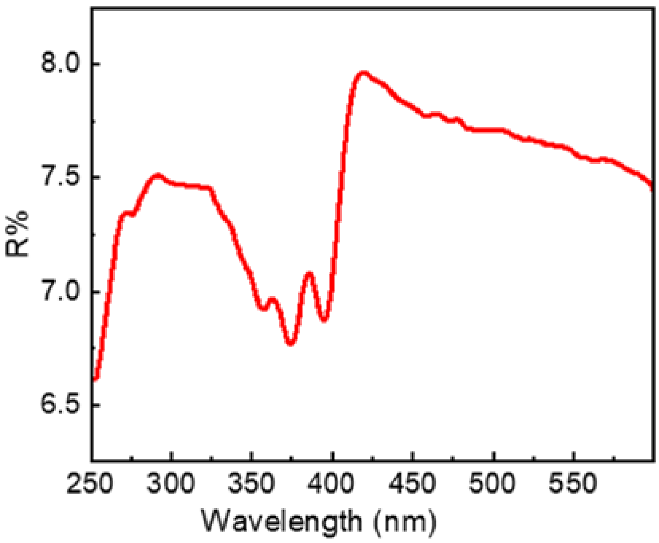

Supplementary Fig. 28 Reflectance characteristics of Zn-ADC-DABCO. Steady-state diffuse reflectance spectrum of the Zn-ADC-DABCO thin film. Source data are provided as a Source Data file.

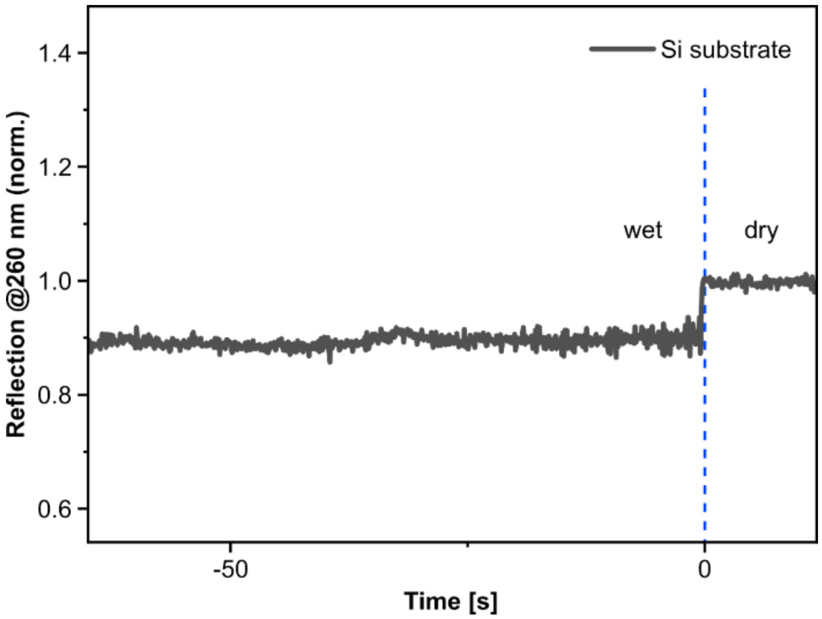

Supplementary Fig. 29 Reflection of Si substrate at 260 nm during ethanol evaporation. In contrast to the Zn-ADC-DABCO thin film, there is only the sudden transition from wet to dry state visible and no increase of the reflection signal right before. The reflection curves at 300 and 500 nm in Fig. 4c closely resemble this step-like behavior from wet to dry state.

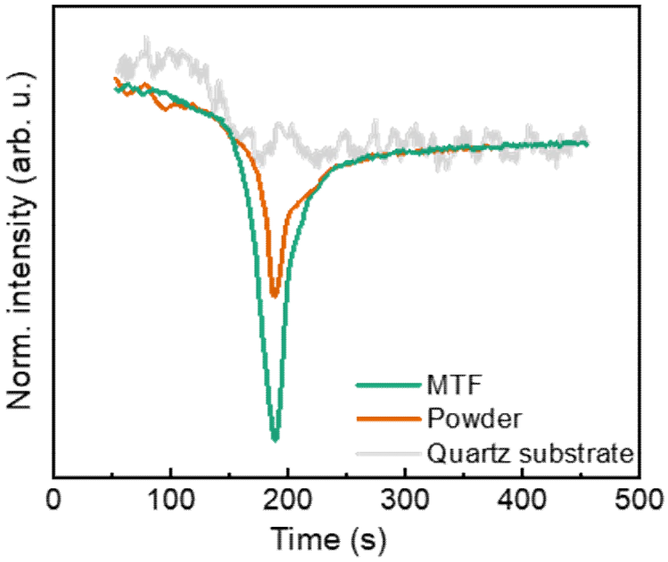

Supplementary Fig. 30 Comparison on absorption of oriented MTF and MOF powder deposited on quartz plate. Absorbance intensity at 365 nm over time. Source data are provided as a Source Data file.

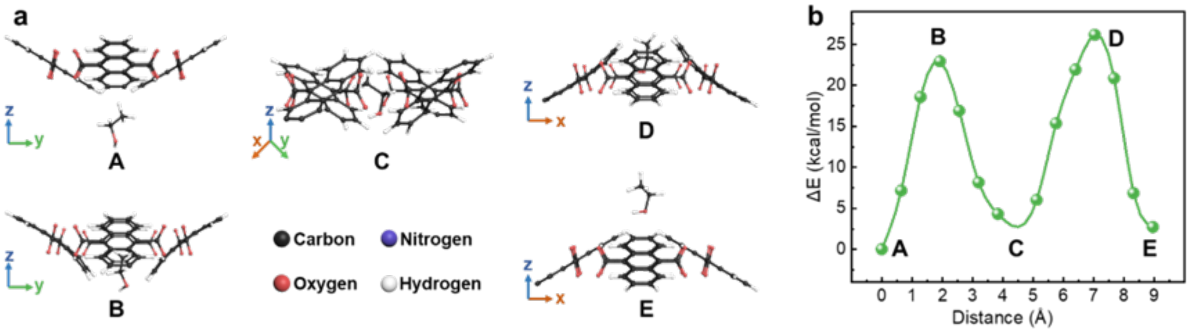

Supplementary Fig. 31 Simulation of single ethanol molecule passes through MOF. (**a**) The process of a single ethanol molecule passes through MOF channel. A: Initial structure of ADC before the ethanol molecule enters. B: Upon entering the channel, the anthracene structure undergoes partial bending and rotation deformation. C: As the ethanol molecule is in the middle region of these four ADC molecules, the structures of ADC exhibit no significant deformation. D: The ethanol molecule is leaving the channel, the anthracene structure undergoes partial bending and rotation deformation again. E: As the molecule exits the channel, the anthracene structure reverts to its original form. For clarity, the Zn ions and DABCO molecules in the unit cell are omitted. **(b)** Rotational energy barrier of the ADC rotor over the ethanol molecule passing through. Source data are provided as a Source Data file.

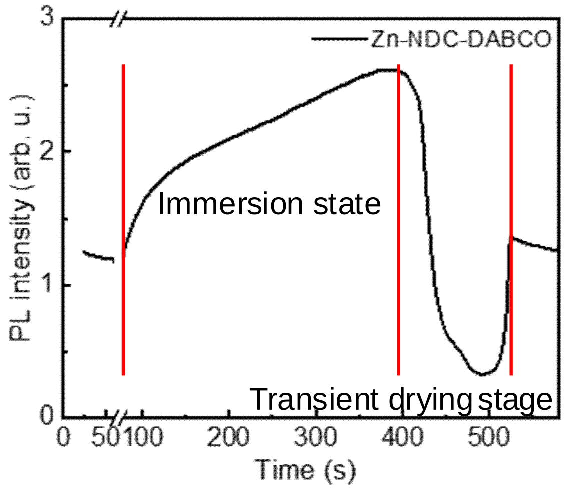

Supplementary Fig. 32 PL evolution of Zn-NDC-DABCO under dropping liquid ethanol. PL intensity kinetics of Zn-ADC-DABCO under excitation of 300 nm LED with ethanol dropping. Source data are provided as a Source Data file.

Supplementary Tables

Supplementary Table 1 Absolute PLQY of various samples upon excitation of 365 nm.

	**PLQY (%)**
ADC in solution[a]	45.5±3.9[d]
MTF[b] dry state	0.667±0.093[d]
MTF light-up state	42.5±3.7[e]
Powder[c] light-up state	20.0±0.9[e]

a, c_{ADC} = 0.1 mM; b, MTF: Zn-ADC-DABCO thin film; c, Powder: Zn-ADC-DABCO powder deposited on quartz plate.; Statistic average from (d) twice and (e) three times.

Supplementary Table 2 Fitted lifetimes of ADC PL decay as monomer in solution and in MOF thin film during ethanol evaporation phases.

**ADC emission phase**	**Short lifetime (weight)**	**Long lifetime (weight)**
MOF dry state	1.89 ns (98 %)	12.63 ns (2 %)
MOF solvation state	2.32 ns (89 %)	8.54 ns (11 %)
MOF flow state	3.39 ns	
Monomer in solution	7.06 ns	

The lifetimes were obtained from mono-exponential (flow state and monomer in solution) and bi-exponential (dry state and solvation state) tail fits of the data shown in **Fig. 4b**.

Supplementary Table 3 Tested conditions for preparation of Zn-ADC-DABCO

	Exp.	Precursor solution				Ethanol (μL)	Droplet volume (μL cm^{-2})	Substrate	Temperature (°C)	FWHM (°)
		$Zn(CH_3OOC)_2{\cdot}2H_2O$	ADC (mmol L^{-1})	DABCO	HCl					
Variation of precursor solution	1	0.02	0.02	0.02	0.02	418	82	Silicon	40	N/A
	2	0.05	0.05	0.05	0.05	418	82	Silicon	40	19.43
	3	0.1	0.1	0.1	0.1	418	82	Silicon	40	14.10
	4	0.15	0.15	0.15	0.15	418	82	Silicon	40	15.81
	5	0.175	0.175	0.175	0.175	418	82	Silicon	40	16.09
	6	0.2	0.2	0.2	0.2	418	82	Silicon	40	18.11
	7	0.25	0.25	0.25	0.25	418	82	Silicon	40	18.55
	8	0.5	0.5	0.5	0.5	418	82	Silicon	40	N/A
	9	0.2	0.2	0.1	0.2	418	82	Silicon	40	N/A
	10	0.2	0.2	0.4	0.2	418	82	Silicon	40	21.46
Variation of the reaction temperature	11	0.2	0.2	0.2	0.2	418	82	Silicon	30	N/A
	12	0.2	0.2	0.2	0.2	418	82	Silicon	50	14.13
	13	0.2	0.2	0.2	0.2	418	82	Silicon	60	13.32
	14	0.2	0.2	0.2	0.2	418	82	Silicon	70	15.61
	15	0.2	0.2	0.2	0.2	418	82	Silicon	80	15.94
	16	0.2	0.2	0.2	0.2	418	82	Silicon	90	15.35
	17	0.2	0.2	0.2	0.2	418	82	Silicon	100	18.96
Optimization	18	0.05	0.05	0.05	0.05	418	82	Silicon	45	17.13
	19	0.1	0.1	0.1	0.1	418	82	Silicon	45	17.89
	20	0.15	0.15	0.15	0.15	418	82	Silicon	45	18.27
	21	0.2	0.2	0.2	0.2	418	82	Silicon	45	15.35
	22	0.25	0.25	0.25	0.25	418	82	Silicon	45	15.57
	23	0.05	0.05	0.05	0.05	418	82	Silicon	50	15.47
	24	0.1	0.1	0.1	0.1	418	82	Silicon	50	14.12

	Exp.	Precursor solution				Ethanol (μL)	Droplet volume (μL cm^{-2})	Substrate	Temperature (°C)	FWHM (°)
		$Zn(CH_3OOC)_2 \cdot 2H_2O$	ADC (mmol L^{-1})	DABCO	HCl					
	25	0.15	0.15	0.15	0.15	418	82	Silicon	50	13.60
	26	0.25	0.25	0.25	0.25	418	82	Silicon	50	15.02
	27	0.05	0.05	0.05	0.05	418	82	Silicon	55	12.25
	28	0.1	0.1	0.1	0.1	418	82	Silicon	55	16.04
	29	0.15	0.15	0.15	0.15	418	82	Silicon	55	16.68
	30	0.2	0.2	0.2	0.2	418	82	Silicon	55	14.99
	31	0.25	0.25	0.25	0.25	418	82	Silicon	55	13.92
	32	0.05	0.05	0.05	0.05	418	82	Silicon	60	13.85
	33	0.1	0.1	0.1	0.1	418	82	Silicon	60	20.96
	34	0.15	0.15	0.15	0.15	418	82	Silicon	60	17.40
	35	0.25	0.25	0.25	0.25	418	82	Silicon	60	23.62
Variation of droplet volume	36	0.15	0.15	0.15	0.15	10	10	Silicon	50	N/A
	37	0.15	0.15	0.15	0.15	20	10	Silicon	50	N/A
	38	0.15	0.15	0.15	0.15	30	10	Silicon	50	N/A
	39	0.15	0.15	0.15	0.15	40	10	Silicon	50	N/A
	40	0.15	0.15	0.15	0.15	80	10	Silicon	50	N/A
	41	0.15	0.15	0.15	0.15	82	82	Silicon	50	24.51
	42	0.15	0.15	0.15	0.15	164	82	Silicon	50	20.49
	43	0.15	0.15	0.15	0.15	328	82	Silicon	50	17.19
Variation of solution volume	44	0.15	0.15	0.15	0.15	30	30	Silicon	50	N/A
	45	0.15	0.15	0.15	0.15	60	30	Silicon	50	N/A
	46	0.15	0.15	0.15	0.15	100	30	Silicon	50	N/A
	47	0.15	0.15	0.15	0.15	200	30	Silicon	50	N/A
	48	0.15	0.15	0.15	0.15	836	30	Silicon	50	15.36
	49	0.15	0.15	0.15	0.15	1254	30	Silicon	50	15.40
	50	0.15	0.15	0.15	0.15	1672	30	Silicon	50	15.48

	Exp.	Precursor solution				Ethanol (μL)	Droplet volume (μL cm^{-2})	Substrate	Temperature (°C)	FWHM (°)
		$Zn(CH_3OOC)_2{\cdot}2H_2O$	ADC (mmol L^{-1})	DABCO	HCl					
Homogeneity	51-1	0.15	0.15	0.15	0.15	1672	30	Silicon	50	15.91
	51-2	0.15	0.15	0.15	0.15	1672	30	Silicon	50	17.30
	51-3	0.15	0.15	0.15	0.15	1672	30	Silicon	50	14.92
	51-4	0.15	0.15	0.15	0.15	1672	30	Silicon	50	14.76
Variation of substrate	52	0.15	0.15	0.15	0.15	418	30	Quartz	50	19.89
	53	0.15	0.15	0.15	0.15	418	30	TiO_2@Si	50	16.59
	54	0.15	0.15	0.15	0.15	418	30	Gd_2O_3@Si	50	18.99
	55	0.15	0.15	0.15	0.15	418	30	Ag@Si	50	17.60
	56	0.15	0.15	0.15	0.15	418	30	ZnO@Si	50	17.07

Supplementary Table 4 Tested conditions for preparation of Zn-ADC-BPy

Exp.	Precursor solution				Ethanol (μL)	Droplet volume (μL cm^{-2})	Substrate	Temperature (°C)	FWHM (°)
	$Zn(CH_3OOC)_2 \cdot 2H_2O$	ADC (mmol L^{-1})	BPy	HCl					
1	0.2	0.2	0.05	0.2	418	82	Silicon	50	17.36
2	0.2	0.2	0.1	0.2	418	82	Silicon	50	17.91
3	0.2	0.2	0.2	0.2	418	82	Silicon	50	23.26

References

1. Fischer, J. C., *et al.* Giwaxs characterization of metal-organic framework thin films and heterostructures: Quantifying structure and orientation. *Adv. Mater. Interfaces* **10**, 2202259 (2023).
2. Jiang, Z.: A matlab toolbox for grazing-incidence x-ray scattering data visualization and reduction, and indexing of buried three-dimensional periodic nanostructured films. *J. Appl. Crystallogr.* **48**, 917-926 (2015).
3. Schrode, B., *et al.*: A comprehensive software tool for geometry-independent grazing-incidence x-ray diffraction data analysis and pole-figure calculations. *J. Appl. Crystallogr.* **52**, 683-689 (2019).
4. Hirai, K., *et al.* Sequential functionalization of porous coordination polymer crystals. *Angew. Chem. Int. Ed.* **50**, 8057-8061 (2011).
5. Rivnay, J., *et al.* Quantitative determination of organic semiconductor microstructure from the molecular to device scale. *Chem. Rev.* **112**, 5488-5519 (2012).
6. Tan, W. L., McNeill, C. R. X-ray diffraction of photovoltaic perovskites: Principles and applications. *Appl. Phys. Rev.* **9**, 021310 (2022).
7. Mahmood, A., Wang, J. L. A review of grazing incidence small- and wide-angle x-ray scattering techniques for exploring the film morphology of organic solar cells. *Sol. Rrl* **4**, 2000337 (2020).
8. Hammond, M. R., *et al.* Molecular order in high-efficiency polymer/fullerene bulk heterojunction solar cells. *Acs Nano* **5**, 8248-8257 (2011).
9. Sauerbrey, G. Verwendung von schwingquarzen zur wagung dunner schichten und zur mikrowagung. *Z Phys* **155**, 206-222 (1959).
10. Oldenburg, M., *et al.* Enhancing the photoluminescence of surface anchored metal-organic frameworks: Mixed linkers and efficient acceptors. *Phys. Chem. Chem. Phys.* **20**, 11564-11576 (2018).
11. Köhler, A., Bässler, H. *Electronic processes in organic semiconductors: An introduction*. John Wiley & Sons, 2015.
12. Kühne, T. D., *et al.* Cp2k: An electronic structure and molecular dynamics software package - quickstep: Efficient and accurate electronic structure calculations. *J. Chem. Phys.* **152**, 194103 (2020).
13. VandeVondele, J., *et al.* Quickstep: Fast and accurate density functional calculations using a mixed gaussian and plane waves approach. *Comput. Phys. Commun.* **167**, 103-128 (2005).
14. Henkelman, G., Uberuaga, B. P., Jónsson, H. A climbing image nudged elastic band method for finding saddle points and minimum energy paths. *J. Chem. Phys.* **113**, 9901-9904 (2000).
15. Grimme, S., Bannwarth, C., Shushkov, P. A robust and accurate tight-binding quantum chemical method for structures, vibrational frequencies, and noncovalent interactions of large molecular systems parametrized for all spd-block elements (z= 1–86). *J. Chem. Theory Comput.* **13**, 1989-2009 (2017).
16. Grimme, S., Antony, J., Ehrlich, S., Krieg, H. A consistent and accurate ab initio parametrization of density functional dispersion correction (dft-d) for the 94 elements h-pu. *J. Chem. Phys.* **132**, 154104 (2010).
17. Grimme, S., Ehrlich, S., Goerigk, L. Effect of the damping function in dispersion corrected density functional theory. *J Comput. Chem.* **32**, 1456-1465 (2011).
18. Gao, Q., *et al.* Tuning the formations of metal-organic frameworks by modification of ratio of reactant, acidity of reaction system, and use of a secondary ligand. *Cryst. Growth Des.* **12**, 281-288 (2012).

19. Goswami, S., *et al.* Toward ideal metal-organic framework thin-film growth via automated layer-by-layer deposition: Examples based on perylene diimide linkers. *Chem. Mater.* 9446-9454 (2022).
20. Tan, K., *et al.* Stability and hydrolyzation of metal organic frameworks with paddle-wheel sbus upon hydration. *Chem. Mater.* **24**, 3153-3167 (2012).
21. Tan, K., *et al.* Water interactions in metal organic frameworks. *Crystengcomm* **17**, 247-260 (2015).
22. Jasuja, H., Huang, Y. G., Walton, K. S. Adjusting the stability of metal-organic frameworks under humid conditions by ligand functionalization. *Langmuir* **28**, 16874-16880 (2012).
