# Supplementary material for: Turn-on luminescence from molecular rotor realignment in metal-organic framework thin films
Source: Nat Commun. 2026 Mar 14;17:3969. doi: 10.1038/s41467-026-70551-8 (PMC13133237; doi:10.1038/s41467-026-70551-8)
Supplement: Supplementary file 4 — Description of Additional Supplementary Files [file 41467_2026_70551_MOESM4_ESM.pdf]

Description of Additional Supplementary Files

Supplementary Data 1

Description: Single ethanol molecule passes through MOF channel.

Supplementary Data 2

Description: Continuous evaporation of ethanol flow through MOF channel.

Supplementary Data 3

Description: ADC molecule rotation is induced by ethanol flow passes from both sides.

Supplementary Movie 1

Description: MOF coating sieve indicates methanol evaporation.
