# Supplementary figures and images for: Turn-on luminescence from molecular rotor realignment in metal-organic framework thin films
Source: Nat Commun. 2026 Mar 14;17:3969. doi: 10.1038/s41467-026-70551-8 (PMC13133237; doi:10.1038/s41467-026-70551-8)

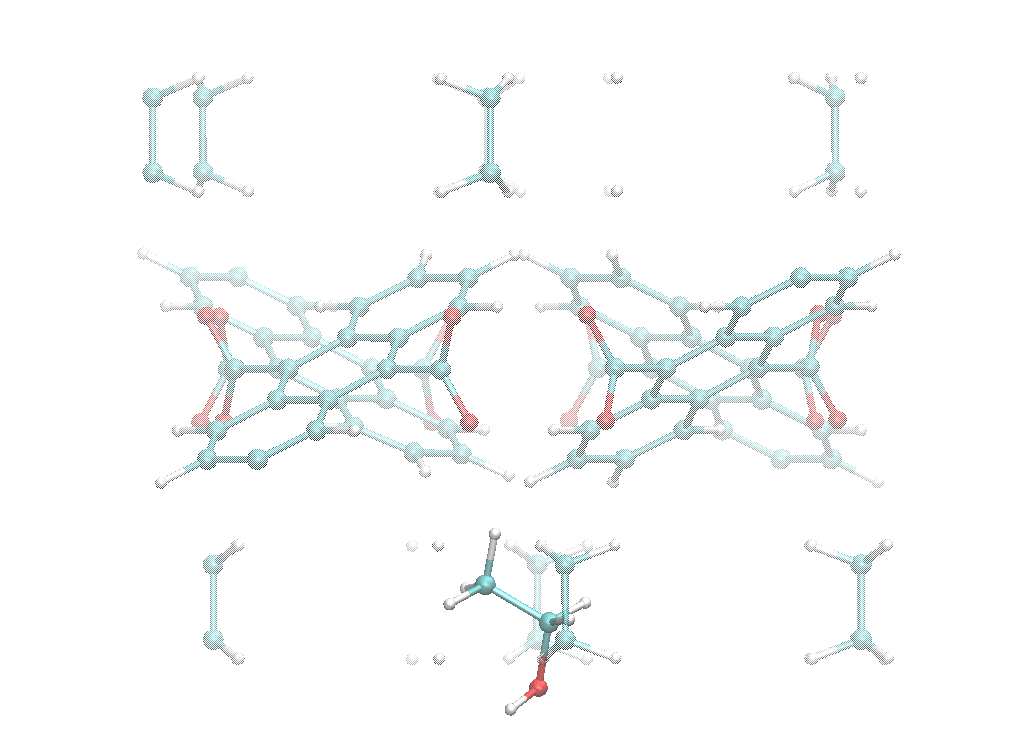

Supplement: Supplementary file 5 — Supplementary Data 1 [file 41467_2026_70551_MOESM5_ESM.gif]

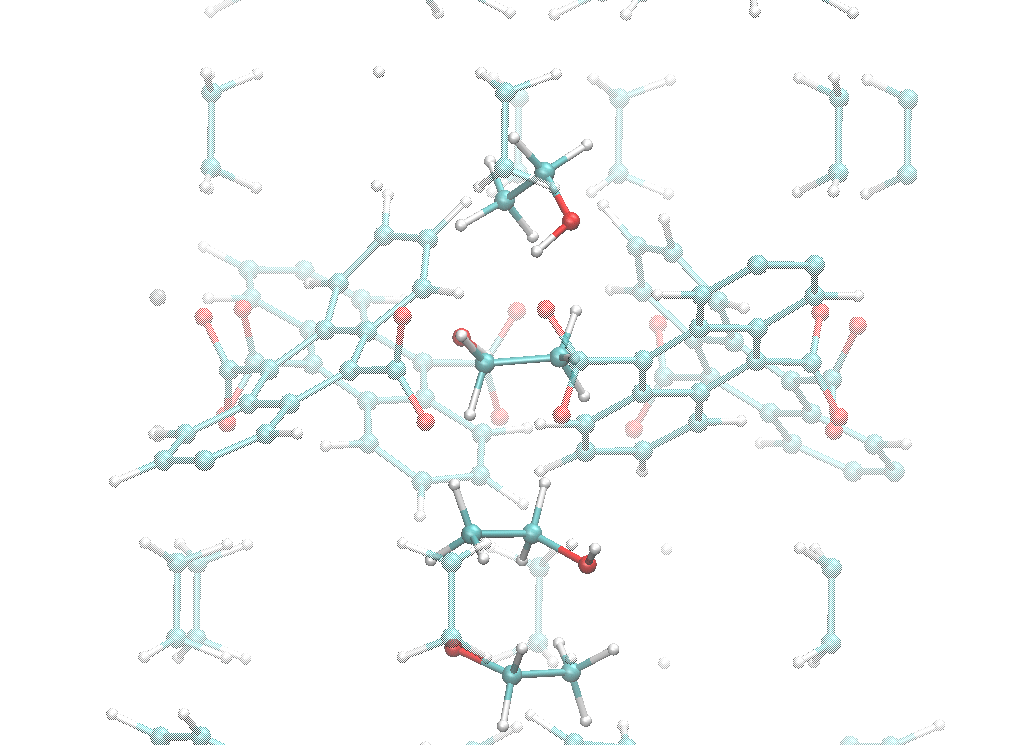

Supplement: Supplementary file 6 — Supplementary Data 2 [file 41467_2026_70551_MOESM6_ESM.gif]

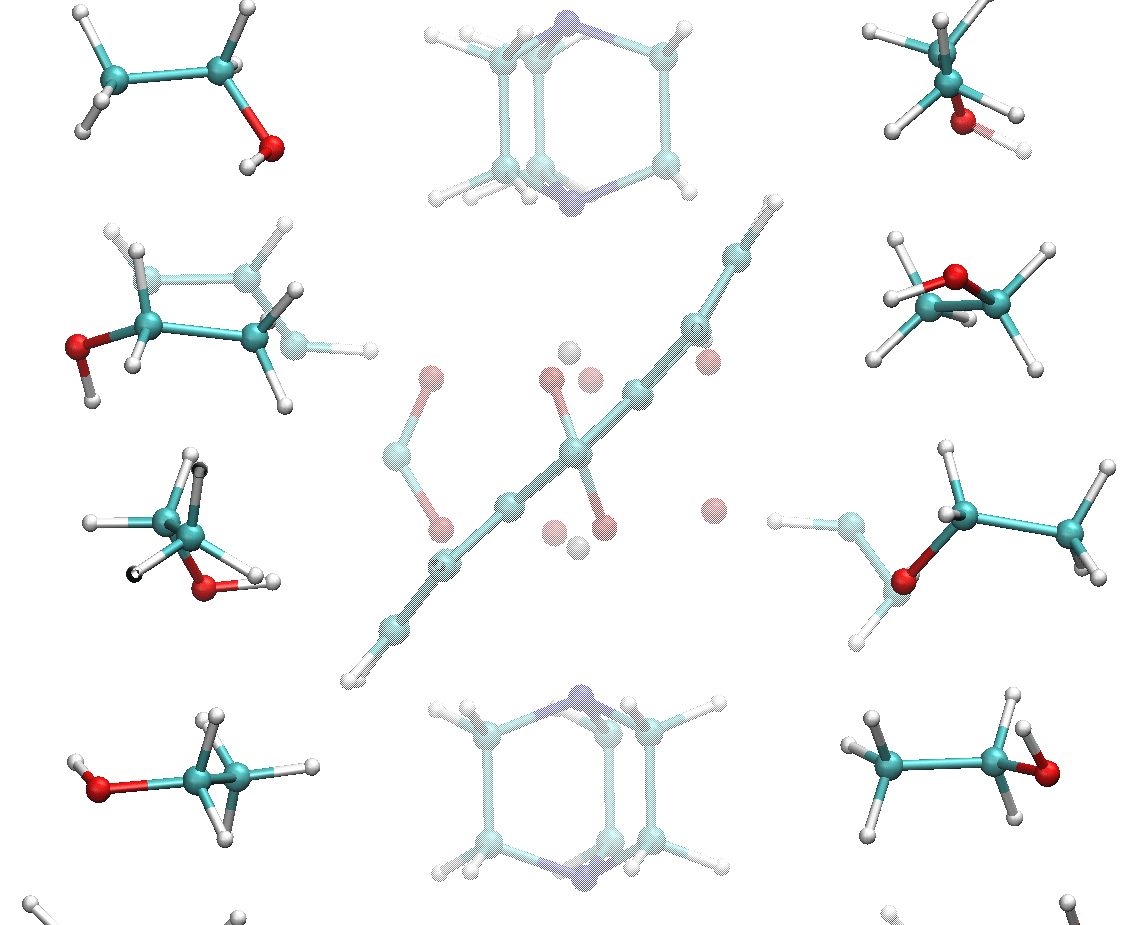

Supplement: Supplementary file 7 — Supplementary Data 3 [file 41467_2026_70551_MOESM7_ESM.gif]
